# Use of NSAIDs and acetaminophen and risk of spontaneous intestinal perforations in premature infants: a systematic review and meta-analysis

**DOI:** 10.3389/fped.2024.1450121

**Published:** 2024-11-22

**Authors:** Jo-Anna B. J. Hudson, Wardha Shabbir, Lamia M. Hayawi, Monica Lik Man Chan, Nicholas Barrowman, Lindsey Sikora, Emanuela Ferretti

**Affiliations:** ^1^ Department of Pediatrics, Division of Neonatology, Memorial University, St. John's, Newfoundland, NF, Canada; ^2^ Department of OBGYN, Newborn Division, University of Ottawa, The Ottawa Hospital, Ottawa, ON, Canada; ^3^ Department of Pediatrics, Division of Neonatology, University of Ottawa, Children's Hospital of Eastern Ontario, Ottawa, ON, Canada; ^4^ Department of Family Medicine, Michigan State University, Jackson, MI, United States; ^5^ Children's Hospital of Eastern Ontario Research Institute, Ottawa, ON, Canada; ^6^ Health Sciences Library, University of Ottawa, Ottawa, ON, Canada

**Keywords:** spontaneous intestinal perforation (SIP), premature (babies), non-steroidal anti-inflammatory drug (NSAID), systematic review, acetaminophen

## Abstract

**Background:**

Acquired spontaneous intestinal perforation or SIP occurs most commonly in the extremely premature infant population. As the incidence is rising, understanding modifiable factors such as common medication exposures becomes important for individualizing care.

**Methods:**

The primary outcome was SIP in premature infants with exposure to indomethacin, ibuprofen, or acetaminophen. The systematic review and meta-analysis were conducted following the Cochrane methodology and PRISMA guidelines.

**Results:**

The point estimates of three RCTs showed an increase in the risk of SIP with indomethacin exposure compared to no medication, the pooled estimate was not statistically significant. There is no statistically significant association between the risk of SIP for indomethacin with treatment use over prophylactic use and when holding feeds. Ibuprofen conferred less risk than indomethacin, and its route of administration did not alter the risk profile. There was not enough evidence to draw conclusions about the risk of SIP and acetaminophen exposure.

**Conclusion:**

In studies of infants exposed to either indomethacin or ibuprofen in the last 40 years, the incidence of SIP is still commonly within 2–8%. Moving forward modifiable factors such as medication exposure will help guide care to minimize risk where possible.

**Systematic Review Registration:**

https://www.crd.york.ac.uk/, PROSPERO (CRD42017058603).

## Introduction

Spontaneous intestinal perforation (SIP) is becoming the most prevalent acquired neonatal intestinal disease in extreme prematurity, outpacing necrotizing enterocolitis (NEC), the former front runner (1, 2). Understanding the architecture of SIP permits analysis and characterization of its relationship to neonatal medications such as non-steroidal anti-inflammatory drugs (NSAIDs) and acetaminophen.

SIP is the localized perforation of the intestine without clinical or histopathological signs of NEC (3). It can be congenital or acquired. Congenital SIP is the rare absence of the intestinal muscularis interna in later gestation infants (4). In acquired SIP, the muscularis layer is present but becomes contracted in the extremely premature and extremely low birth weight populations (4). The clinical hallmark of SIP is the overall stability of the infant (3). SIP presents between 0 and 10 days of life with a shiny distended abdomen, often bluish in color, without the presence of loops (1). On radiography, the abdomen is gasless, and pneumoperitoneum is present without pneumatosis (1). The process is self-limiting with rare cases of recurrence or strictures (5). SIP predominately occurs on the antimesenteric border in the distal ileum (3, 5). On histopathological examination, there is focal hemorrhagic necrosis with a defined border surrounded by healthy appearing bowel (3). The intestinal muscularis propria is thin, with thin-walled vessels in the adjacent submucosa.

There are numerous postulated risk factors for SIP. One of them involves an ischemic hit to the watershed blood supply to the distal ileum (e.g., low APGAR scores, stress, hypoxia, shock, or microemboli), but this does not account for SIP occurring in other locations (3, 6). The patent ductus arteriosus (PDA) is felt to contribute to the risk, due to the decrease in mesenteric blood supply secondary to diastolic steal (7). Other risk factors include prematurity, low birth weight, infection, feeding regime, and antenatal/postnatal medications (2, 3). To understand the potential associations, it is paramount to review the underlying physiology.

Antenatal intestinal growth is driven by insulin-like growth factor -I (IGF-I) (5). Levels are decreased in lower gestation and low birth weight infants, resulting in thinner bowel walls (1, 5). Thicker meconium, associated with premature bowel hypomotility, can increase pressure on the thin intestinal walls (3). Postnatal medications further derange this physiology. NSAIDs including indomethacin and ibuprofen are cyclooxygenase inhibitors that competitively bind to block prostaglandin synthesis (8). In human fetal tissue, indomethacin has pernicious effects occurring at the genomic level disrupting critical metabolic pathways known to elicit a protective response to oxidative stress (9). Additionally, indomethacin reduces the amount of nitric oxide synthase and its precursor arginine during mid-gestation in the human gut (1, 10). Reduced nitric oxide synthase exacerbates intestinal dysmotility. Decreased arginine has deleterious effects on bowel wall tight junctions, disrupting the intestinal barrier, and resulting in increased bacterial translocation (9, 10). The detrimental effects of indomethacin on the gut are compounded when taken concurrently with steroids resulting in depletion of all nitric oxide synthase isoforms, intestinal mucosal hyperplasia, and submucosal thinning (1, 5). When medications are stopped and nitric oxide levels normalize, bowel motility returns, placing pressure on the weakened structure culminating in the potential for SIP. Ibuprofen is another NSAID, with milder effects on cerebral, renal, and mesenteric blood flow compared to indomethacin (11). Acetaminophen also inhibits prostaglandin synthesis through a selective mechanism that does not have the same peripheral vasoconstriction as NSAIDs (8).

The incidence of SIP is increasing, and determining risk factors is cardinal when balancing minimizing peril with maximizing care for this fragile population. The goal of this systematic review was to provide a current analysis of the association of SIP with exposure to indomethacin, ibuprofen, and acetaminophen.

## Methods

### Protocol registration

The protocol was registered on PROSPERO (https://www.crd.york.ac.uk/prospero/display_record.php?ID=CRD42017058603). The strategy followed methods outlined in the Cochrane Handbook for Systematic Reviews, as well as the Preferred Reporting Items for Systematic Reviews and Meta-Analyses (PRISMA) Statement (12, 13). Any differences from the published protocol were decided as a group and outlined in Supplementary Table 1 (14).

### Eligibility of studies

The study population was neonates born less than 37 weeks or birth weight less than 2.5 kg. The intervention was exposure to one of the following medications: indomethacin, ibuprofen, or acetaminophen.

The following study designs in any language worldwide were included: randomized controlled trials, clinical trials (for unpublished clinical trials, the corresponding authors were contacted), cohort studies with a control group, and case–control studies.

Eligible studies were required to meet the above criteria as well as the definition of SIP listed below.

### Outcomes

#### Definition of SIP

SIP became a separate recognized entity in 2002 by the National Institute for Child Health and Human Development (NICHD) (4). Historically, SIP and NEC were grouped together with intestinal perforations occurring in both within the same high-risk population, until the 2002 NICHD definition. The timeframe for this systematic review predates the established 2002 definition. To ensure clarity, the definition of SIP in our study was based on the following timeline:

1. Before the 2002 NICHD definition, SIP was defined by either radiological (pneumoperitoneum, intramural echogenicity, and echogenic extramural material on abdominal ultrasonography as pathognomonic signs with no evidence of pneumatosis intestinalis), histopathologic evidence (focal hemorrhagic necrosis, possible hypoplasia of the muscularis layer, and thinning of the submucosa) or surgical evidence of isolated intestinal perforation in the absence of histopathological features of necrotizing enterocolitis (3, 15, 16).
2. After the 2002 NICHD definition, if SIP was listed as an outcome, it was accepted.

#### Primary outcome

The primary outcome was SIP with comparisons between those exposed to one of the listed medications to either no medication, to an alternate medication from that list, or a prophylactic vs. treatment regimen comparison. In this systematic review, articles designed to compare prophylactic to treatment regimens were pooled regardless of the dose or underlying condition being prevented/treated. Prophylactic medication was given within the first 24 h of life, and treatment was given later than 24 h of life for diagnosed PDA management.

#### Secondary outcomes

The secondary outcomes (with definitions) were NEC (Bell stage 2 or greater), oliguria (<1 ml/kg/h of urine output), and neonatal death prior to discharge (17). If a study did not meet the definition, the outcome was excluded.

#### Additional clinical questions and characteristics

The relationship of SIP with medication exposure through the lens of feeding regimes and the ibuprofen route of administration was also explored. Additional characteristics were captured (with definitions) including intraventricular hemorrhage (IVH) (any grade using Papile criteria of IVH), retinopathy of prematurity (ROP) (any stage using the International Classification of ROP), and bronchopulmonary dysplasia (BPD) (requirement of oxygen and/or positive pressure at 36 weeks postmenstrual age or at time of discharge if prior to 36 weeks) (18–20). Antenatal medication information including steroids, magnesium sulfate, and indomethacin, as well as postnatal steroids (not for blood pressure support), was collected.

### Search strategy and study selection

A search strategy was developed by a medical librarian in Medline and then translated into the other databases (Embase Classic and OVID, PubMed, LILAC, ScIELO, and Cochrane Central) to retrieve articles. All databases were searched from their dates of inception to 13 November 2016, updated on 19 February 2021, 4 May 2022, and lastly on 30 September 2022 (Supplementary Data Sheet 1). All references were entered into Endnote for processing (21). The initial duplicate screening was performed using Covidence (22). On 9 September 2023, two additional search strategies were performed: systematic review snowballing and systematic review reference check (details in Supplementary Data Sheet 1) (23). Two reviewers independently screened abstracts and full texts of all retrieved articles, and any conflicts were resolved by the biostatisticians.

### Risk of bias appraisal

Two authors conducted risk of bias assessment independently for all included studies. The Cochrane Risk of Bias tool-II (RoB 2) was used for the risk of bias assessment for randomized trials, and the ROBINS-I tool was used for the assessment of non-randomized trials (24, 25). The Cochrane methodology was followed for missing data (26). Any inconsistencies were resolved with the assistance of the biostatisticians.

### Data synthesis strategy

#### Data extraction

All data were extracted and incorporated into Excel (Supplementary Data Sheet 2). This process was conducted independently by two reviewers. Any inconsistencies or queries were resolved by the biostatisticians.

#### Data analysis

All statistical analyses were conducted using R statistical software (version 4.2.1) (27) with the “meta” package (28). Randomized control trial (RCT) studies and non-RCT studies were grouped and analyzed separately. Odds ratios were pooled for comparative studies using the DerSimonian and Laird random-effects model for meta-analysis (29). In supplementary analyses, incidence proportions were pooled for one group of studies using a random-effects logistic regression model (30). Heterogeneity in the study estimates were calculated using the *I*^2^ statistic (31). When *I*^2^ was greater than 75%, the heterogeneity was considered high, and pooled estimates were not reported. Qualitative synthesis with either narrative description or tabular representation was presented when studies could not be quantitatively combined due to unacceptable heterogeneity or missing data precluding meta-analysis.

Funnel plots to assess publication bias were not performed as they did not meet the threshold of having at least 10 comparative studies of odds ratios of SIP pooled in a meta-analysis (32).

For both analyses, the risk of SIP with indomethacin exposure was stratified by feeding regimes, and for the risk of SIP with ibuprofen exposure stratified by route of administration, a meta-analysis was conducted pooling odds ratio estimates from comparative studies. Additionally, these questions were examined using data from studies that presented data for one group, and a test for subgroup analysis comparing the pooled incidence ratios estimate for each group was conducted.

A subgroup analysis in addition to Fisher's exact test was performed to compare the SIP proportion for patients taking ibuprofen (not enough studies for Indomethacin) in studies in the last decade compared to prior.

## Results

### Study selection

**Figure 1:**
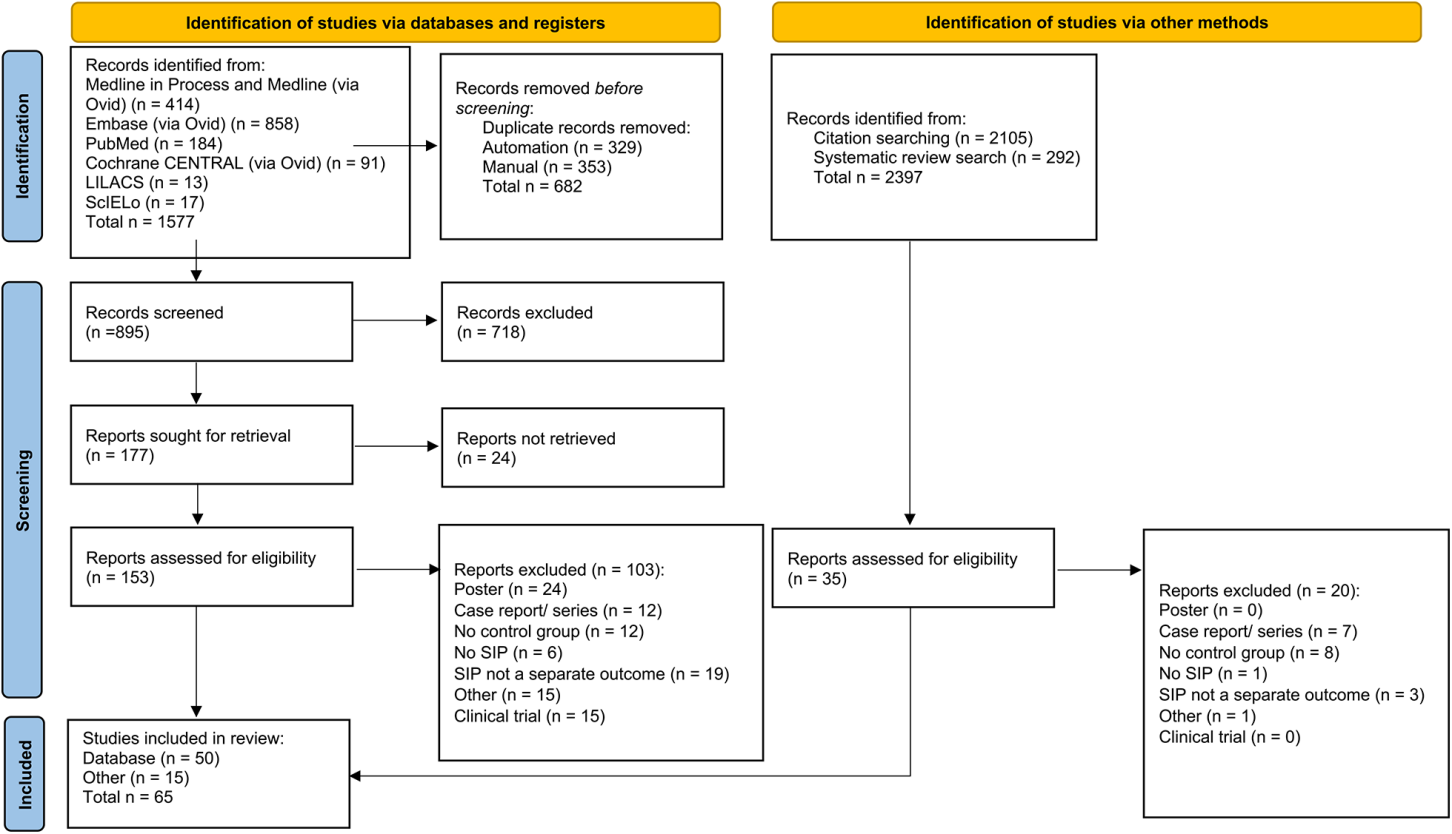
PRISMA 2020 flow diagram for new systematic reviews which included searches of databases, registers, and other sources of study selection process (33).

The database search identified 1,577 articles. From this, 153 articles were assessed for eligibility, resulting in the inclusion of 45 articles. Through reference searching and systematic review searching, an additional 2, 397 articles were identified giving an additional 20 articles for inclusion bringing the included article total to 65 (Figure 1). Supplementary Figure 1 contains the PRISMA 2020 flow charts for each search. Supplementary Table 2 contains the reasons for article exclusion after full-text screening. A study by Ghanem et al. was not included in the meta-analysis as it did not fit the criteria of RCT or cohort/case–control study (34).

### Study characteristics

**Figure 2:**
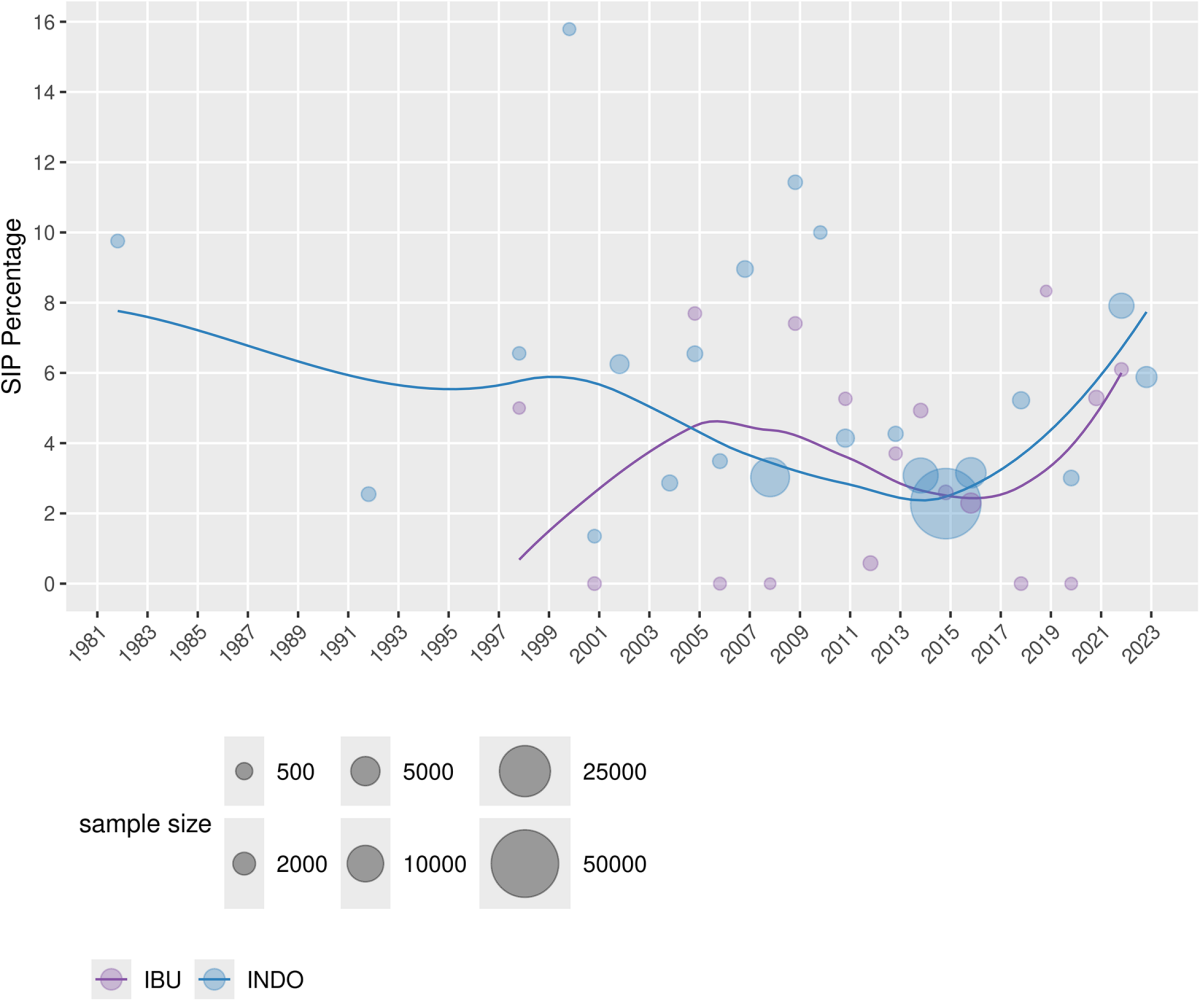
Percentage of SIP in the included studies across the years for both INDO and IBU, with a smoothing line (weighted by sample size).

Table 1 displays the characteristics of the RCT and non-RCT studies that were included in the analyses. There were 25 RCT studies, 1 non-randomized trial, 31 cohort studies, and 8 case–control studies. Thirty-six, 10, and 1 study looked at indomethacin, ibuprofen, and acetaminophen, respectively, as the sole medication. There were 18 studies that used more than one medication of interest. As indomethacin and ibuprofen were the most studied medications, the incidence proportion of SIP over time per medication was graphed (Figure 2). It demonstrated a decreasing reported percentage over time until the last few years when it subjectively began to rise.

**Table 1:** Characteristics of studies included in data analysis.

Year	Author	Country	Study design	Comparison	Medication	SIP	NEC	Death before discharge	AKI
Randomized control trials									
1991	Rennie (35)	United Kingdom	RCT	Prolonged low-dose INDO for PDA management	INDO	Y	N	Y	N
1997	Van Overmeire (36)	Belgium	RCT	INDO versus IBU for PDA closure	INDO and IBU	Y	N	Y	N
2000	Van Overmeire (37)	Belgium	RCT	INDO versus IBU for PDA closure	INDO and IBU	Y	Y	N	N
2001	Schmidt (38)	Canada	RCT	Long-term effects of INDO prophylaxis	INDO	Y	Y	N	Y
2001	Stark (39)	United States of America	RCT	Effects of early steroids	INDO	Y	N	N	N
2001	Van Overmeire (40)	Belgium	RCT	Early versus late INDO for PDA	INDO	Y	N	N	N
2003	Lee (41)	Singapore	RCT	Low versus regular dose INDO for PDA treatment	INDO	Y	Y	Y	Y
2004	Gournay (42)	France	RCT	IBU for PDA management	IBU	Y	N	N	N
2004	Watterberg (43)	United States of America	RCT	Early hydrocortisone versus placebo	INDO	Y	N	N	N
2005	Adamska (44)	Poland	RCT	INDO versus IBU for PDA treatment	INDO and IBU	Y	Y	N	N
2005	Gimeno Navarro (45)	Spain	RCT	INDO versus IBU for PDA treatment	INDO and IBU	Y	N	N	Y
2007	Aly (46)	United States of America	RCT	IBU versus INDO for PDA treatment	INDO and IBU	Y	N	N	N
2008	Salama (47)	Qatar	RCT	IBU versus INDO for PDA treatment	INDO and IBU	Y	Y	Y	N
2008	Su (48)	China	RCT	INDO versus IBU for early PDA treatment	INDO and IBU	Y	N	Y	Y
2009	Attridge (49)	United States of America	RCT	Beta-natriuretic peptide to guide PDA treatment	INDO	Y	Y	Y	N
2011	Gokman (50)	Turkey	RCT	IBU for PDA administration route	IBU	Y	N	N	N
2012	Erdeve (51)	Turkey	RCT	IBU for PDA administration route	IBU	Y	N	N	N
2012	Sosenko (52)	United States of America	RCT	Early versus expectant management of PDA	IBU	Y	Y	Y	N
2013	Dani (53)	Italy	RCT	Paracetamol versus IBU for PDA treatment	IBU and ACETA	Y	N	N	Y
2013	Kanmaz (54)	Turkey	RCT	IBU for PDA prophylaxis	IBU	Y	N	N	N
2014	Lago (55)	Italy	RCT	Continuous or bolus IBU for PDA treatment	IBU	Y	N	Y	Y
2017	Demir (56)	Turkey	RCT	IBU for PDA administration route	IBU	Y	N	N	N
2018	Hochwald (57)	Israel	RCT	PDA treatment either IBU and ACETA or just IBU	IBU and ACETA	Y	Y	N	Y
2019	El-Farrash (58)	Egypt	RCT	Paracetamol versus IBU for PDA closure	IBU and ACETA	Y	N	N	N
2021	Davidson (59)	United States of America	RCT	INDO versus ACETA for PDA treatment	INDO AECTA	Y	Y	Y	N
Non-randomized control trial studies									
1981	Nagaraj (60)	United States of America	Retrospective cohort	GI complications post PDA treatment with indomethacin	INDO	Y	Y	Y	N
1991	Rajadurai (61)	Australia	Retrospective cohort	INDO for PDA treatment	INDO	Y	N	N	N
1993	Novack (62)	United States of America	Case–control	Characteristics of SIP	INDO	Y	Y	N	N
1996	Raghuveer (63)	United Kingdom	Case–control	Comparison of intestinal perforation to controls	INDO	Y	N	N	N
1997	Kumar (64)	Australia	Retrospective cohort	Prolonged low-dose INDO for PDA treatment	INDO	Y	Y	N	Y
1999	Gordon (65)	United States of America	Retrospective cohort	INDO and/or early dexamethasone effect on SIP	INDO	Y	N	N	N
1999	Shorter (66)	United States of America	Case–control	INDO related perforations	INDO	Y	N	N	N
2003	O'Donovan (67)	United States of America	Retrospective cohort	GI complications with INDO-treated PDA	INDO	Y	Y	Y	N
2005	Sperandio (68)	Germany	Retrospective cohort	INDO dosing	INDO	Y	N	N	N
2006	Attridge_1 (69)	United States of America	Case–control	Association of SIP and INDO	INDO	Y	N	N	N
2006	Attridge_2 (70)	United States of America	Retrospective cohort	Discharge outcomes of SIP	INDO	Y	Y	N	N
2006	Attridge_3 (71)	United States of America	Case–control	Association of SIP and antenatal steroids	INDO	Y	N	N	N
2006	Kawase (72)	Japan	Retrospective cohort	GI perforations and risk factors	INDO	Y	Y	N	N
2007	Cordero (73)	United States of America	Retrospective cohort	Prophy vs. expectant (if symptomatic PDA were treated)	INDO	Y	Y	N	N
2007	Laughon (74)	United States of America	Retrospective cohort	PDA management strategies	INDO	Y	Y	N	N
2008	Sangem (75)	United States of America	Retrospective cohort	Multiple courses of INDO PDA management	INDO	Y	Y	Y	N
2010	Katakam (76)	United States of America	Retrospective cohort	INDO versus IBU for PDA treatment	INDO and IBU	Y	N	N	N
2010	Sharma (77)	United States of America	Prospective cohort	Pre- and postnatal INDO and gut injury	INDO	Y	Y	N	N
2012	Kaempf (78)	United Kingdom	Retrospective cohort	Less aggressive PDA treatment	INDO	Y	N	N	N
2013	Sivanandan (79)	Canada	Retrospective cohort	INDO versus IBU for PDA treatment	INDO and IBU	Y	Y	N	Y
2013	Wadhawan (80)	United States of America	Prospective cohort	Long-term outcome of SIP with INDO	INDO	Y	N	N	N
2014	Chan (81)	Hong Kong	Retrospective cohort	INDO versus IBU for PDA treatment	INDO and IBU	Y	Y	Y	N
2014	Fisher (82)	United States of America	Prospective cohort	Mortality of SIP	INDO	Y	Y	N	N
2014	Kelleher (83)	United States of America	Retrospective cohort	Prophylactic INDO and enteral feeding	INDO	Y	N	Y	N
2015	Gulack (84)	United States of America	Retrospective cohort	INDO versus IBU	INDO and IBU	Y	N	Y	N
2015	Shah (85)	Canada	Retrospective cohort	Risk factors of SIP for outcome	INDO and IBU	Y	Y	N	N
2017	Luecke (86)	United States of America	Retrospective cohort	ACETA for PDA treatment	ACETA	Y	N	N	N
2017	Stavel (87)	Canada	Retrospective cohort	INDO prophylaxis and feeding on SIP	INDO	Y	Y	Y	N
2017	Vongbhait (88)	United States of America	Case–control	Intestinal perforations compared to controls for risk factors	INDO	Y	Y	N	N
2019	Vaidya (89)	United States of America	Retrospective cohort	ACETA compared to INDO for PDA treatment	INDO and ACETA	Y	N	N	N
2019	Waldovgel (90)	Switzerland	Retrospective cohort	High-dose INDO for PDA	INDO	Y	Y	N	Y
2020	Ndour (91)	France	Retrospective cohort	IBU treatment for PDA	IBU	Y	Y	N	Y
2020	Prasad (92)	United States of America	Case–control	Risk factors for SIP	INDO	Y	N	N	N
2021	Arnautovic (93)	United States of America	Case–control	Risk factors associated with SIP	INDO	Y	N	N	N
2021	Graham (94)	United Kingdom	Retrospective cohort	IBU for PDA closure	IBU	Y	N	N	N
2021	Kandraju (95)	Canada	Retrospective cohort	Antenatal steroids and INDO exposure and SIP	INDO	Y	N	N	N
2021	Zozaya (96)	Canada	Retrospective cohort	Neurodevelopment outcomes of SIP and NEC	INDO and IBU	Y	Y	N	N
2022	Chawla (97)	United States of America	Retrospective cohort	Severe intracranial hemorrhage and INDO	INDO	Y	Y	Y	N
2022	Qureshi (98)	Canada	Retrospective cohort	Effect of INDO on brain and gut injury	INDO	Y	Y	Y	N

RCT, randomized control trial; INDO, indomethacin; IBU, Ibuprofen; ACETA, acetaminophen; Y, yes; N, no; SIP, spontaneous intestinal perforation; NEC, necrotizing enterocolitis; AKI, acute kidney injury; GI, gastrointestinal; PDA, patent ductus arteriosus.

### Meta-analysis

Descriptive statistics and tables of evidence summary are in Supplementary Data Sheet 3.

### Primary outcome—SIP

Table 2 depicts the results for the primary outcome.

**Table 2:** Risk of SIP in premature infants who received NSAID medication combinations for comparison for primary outcome analysis.

Comparison for risk of SIP	Study design	Number of studies	Comparison variable	Total number of infants	Odds ratio	95% confidence interval	*I* ^2^
INDOMETHACIN							
INDO vs. no medication	RCT	3	INDO	1028	1.78	0.73; 4.35	49%
			No medication	755			
INDO at a treatment vs. prophylactic use	RCT	3	INDO Treatment use	10153	0.84	0.58; 1.20	66%
			INDO Prophylactic use	10472			
IBUPROFEN							
IBU vs. no medication	RCT	3	IBU	142	1.90	0.31; 11.75	48%
			No medication	140			
Prevalence of SIP with IBU over time	RCT	9	Before 2012	447	Proportion 0.01	0.00; 0.03	36%
	RCT	8	2012 and after	433	Proportion 0.01	0.00; 0.03	27%
			Test for subgroup differences $\chi_{1}^{2}$ = 0.01 df =1 (*p* = 0.94)				
INDO vs. IBU	RCT	6	INDO	206	1.71	0.66; 4.42	0%
			IBU	210			
	Cohort	5	INDO	5362	1.31	0.89; 1.92	32%
			IBU	1363			

*χ*^2^, Chi square; df, degrees of freedom; *I*^2^, heterogeneity; IBU, ibuprofen; INDO, indomethacin; RCT, randomized control trial; vs., versus.

#### Indomethacin

**Figure 3:**
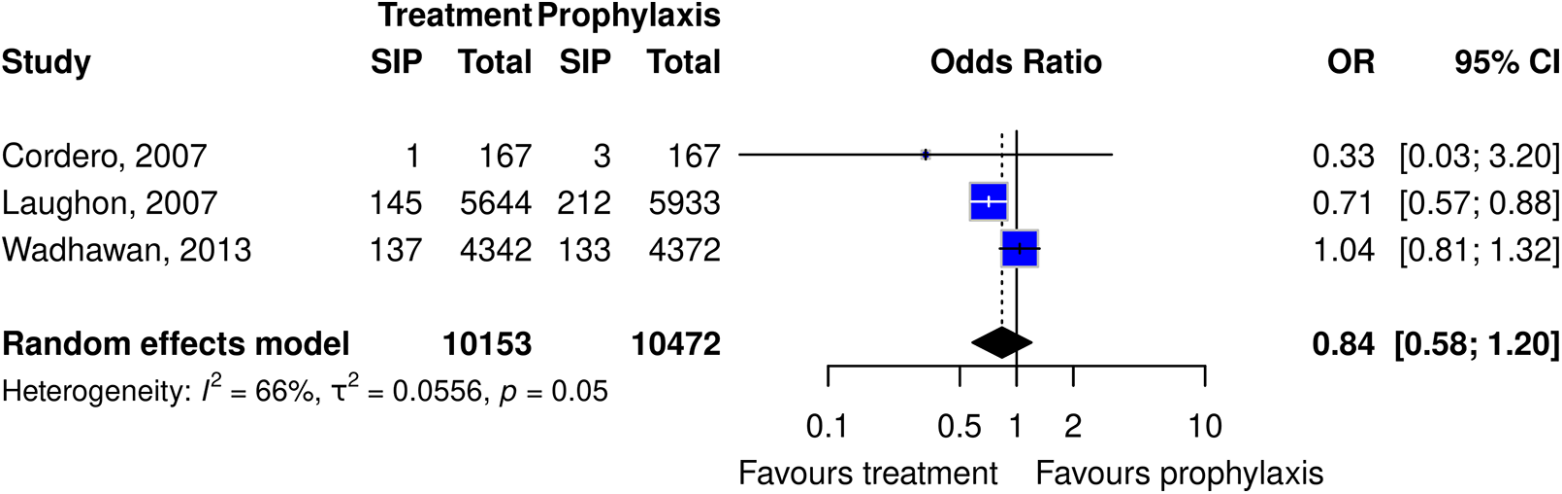
Forest plot of the risk or SIP in premature infants taking indomethacin as a treatment vs. prophylaxis. (All studies are cohort studies).

Several indomethacin comparisons were explored including indomethacin vs. no medication and prophylactic vs. treatment use. The RCT pooled estimate showed no evidence for the risk of SIP in premature infants with indomethacin compared to the no medication group. Three RCTs looked at indomethacin prophylactic vs. treatment use. Of the three, two had larger sample sizes and suggested that indomethacin treatment use may have less risk of SIP compared to prophylactic use; however, the pooled estimate was borderline (Figure 3).

#### Ibuprofen

The association of SIP in premature infants exposed to ibuprofen compared to no medication was examined. Based on RCT data, there was no evidence to support increased odds of SIP in infants taking ibuprofen compared to the no medication group. Given the rise in ibuprofen use over the last decade, a time-based comparison of prevalence was made, comparing the last decade (2012 to present) to the previous (prior to 2012). From this, there was no difference in the prevalence of SIP in premature infants exposed to ibuprofen in the last decade.

#### Indomethacin vs. ibuprofen

Studies designed to compare indomethacin to ibuprofen included six RCT studies and five cohort studies. For both study designs, the pooled estimates did not reach significance; however, the results trended toward less risk of SIP in those taking ibuprofen vs. indomethacin.

#### Acetaminophen

There were significantly fewer articles exploring acetaminophen and SIP with no articles looking at SIP and acetaminophen exposure compared to no medication. Due to the limited number of articles, meta-analysis of the acetaminophen studies was not possible.

### Secondary outcomes

The secondary outcomes explored included the risk of NEC, oliguria, and death before discharge with the same medication exposure combinations as the primary outcome. See Figures S10–S29 in Supplementary Data Sheet 3.

### Additional clinical questions and characteristics

#### Risk of various outcomes with indomethacin exposure stratified by feeding regime

**Figure 4:**
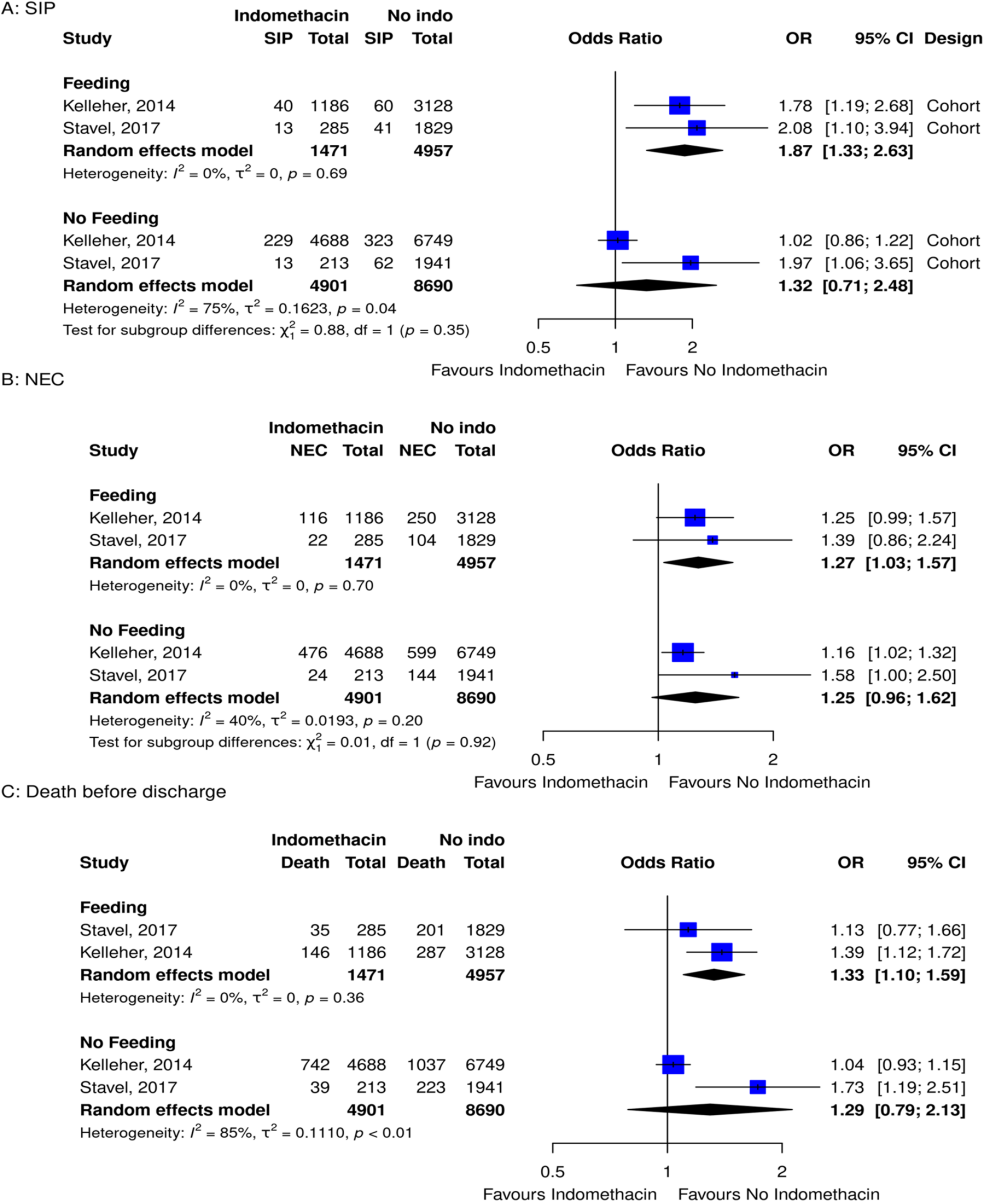
Forest plots for subgroup meta-analyses for the risk of **(A)** SIP, **(B)** NEC, and **(C)** death before discharge grouped by feeding and no feeding groups in infants that took indomethacin vs. no medication.

When layering on the complexity of feeding regimens with medication exposure, there were only studies exploring indomethacin. In analyzing the risk of SIP, NEC, and death before discharge in premature infants with indomethacin exposure—stratified by feeding vs. not feeding during treatment—there were two studies that explored this relationship. It was evident that premature infants on assisted feeding, and taking indomethacin, had a higher risk of SIP, NEC, and death before discharge compared to those who did not take indomethacin (Figure 4).

#### Ibuprofen oral vs. intravenous administration route in premature infants

There are three possible administration routes for ibuprofen: rectal, oral, or intravenous. Rectal administration was excluded as there was only one article, leaving oral and intravenous administration routes for comparison. In a comparative analysis, there were two studies that compared the ibuprofen administration route (IV vs. oral); however, only one study had one case of SIP, the second study had no SIP cases so we could not pool the estimate (Supplementary Data Sheet 3, see Figure S9C). When pooling estimates from non-comparative studies, there were 12 RCTs (8 had IV administration, and 4 had oral administration), we pooled SIP proportions for each route, and a test for subgroup difference showed no difference in the pooled proportion risk of SIP with ibuprofen administration orally vs. intravenous route. (Supplementary Data Sheet 3, see Figure S9B).

#### Additional characteristics

For the incidence of ROP, BPD, and IVH as well as analysis of antenatal medications (steroids, indomethacin, magnesium sulfate) and postnatal steroids, see Supplementary Data Sheet 3, pages 41–46.

### Risk of bias assessment

**Figure 5:**
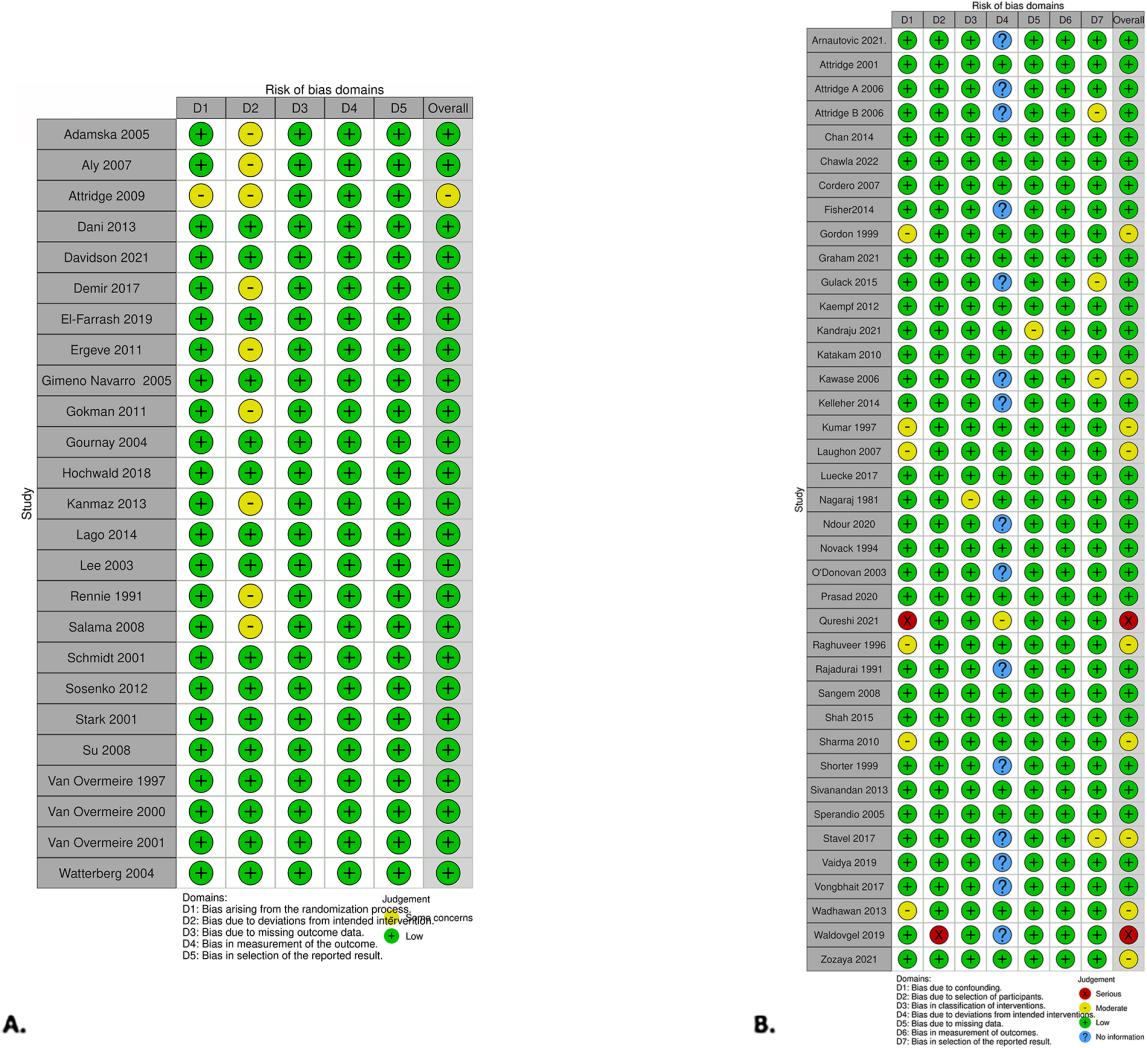
Risk of bias was presented using the risk-of-bias visualization tool. **(A)** RCT studies using RoB2. **(B)** Non-RCT studies using the ROBINS-I tool (99).

The RCT risk of bias assessment revealed one out of the twenty-five studies that showed some concern while the remainder were assessed at low risk for bias (Figure 5). A concern within the RCT studies was not declaring if the analysis was done in an intention-to-treat manner. The study that had some concerns did not provide details about the randomization process (49). The intervention group had the total doses of medication determined by lab values. Measures for concealing groups were not discussed.

Within the non-RCT studies, two showed serious concern, nine had moderate concern, and the remaining were assessed as low risk for bias (Figure 5). Qureshi et al. had serious concerns (98). While exploring infants who had prophylactic indomethacin and outcomes, the authors noted which infants had a PDA but did not adjust for treatment of PDA with indomethacin or comment if it was considered. The number of infants with a PDA was not significantly different between the two groups. Waldvogel et al. also had serious concerns (90). The study's design was to compare standard to high-dose indomethacin for PDAs. All infants received a standard dose of indomethacin, and then when the PDA did not close, they went on to have a high dose, making the direct comparison between the groups unequal.

### Discussion

One in twenty premature infants born at less than 2.5 kg, exposed to indomethacin, are at risk of developing SIP. As the incidence of SIP is on the rise, medication exposure is a modifiable variable, allowing for individualized care. To aid in these crucial decisions, one must look at the medications both in isolation and in the global context within neonatology.

The landscape of neonatology is ever-changing, with the evolution of indomethacin use as an example, initially used for the prevention of IVH and later for PDA management (98, 100, 101). It was the most prevalent medication captured in this review. Over time numerous clinical questions around the use of indomethacin and the risk of SIP have risen including the following: what is the general risk of SIP when exposed to indomethacin, does using it for prophylaxis vs. treatment modalities alter the risk of SIP, and is there an increased risk of SIP when taken indomethacin while feeding compared to feeds being held? We identified three RCT studies looking at SIP with exposure to indomethacin vs. no medication, and together they did not demonstrate an increased risk of SIP when taking indomethacin.

Three different RCTs explored treatment vs. prophylactic indomethacin use. It suggested a trend for a lower risk of SIP with indomethacin treatment compared to prophylaxis use. Moreover, all three RCTs had different clinical designs/questions, but each compared early/prophylactic use within the first 24 h (either for IVH or PDA prevention) and compared it to PDA treatment use after 24 h of life. Given that PDA is a risk factor for SIP, one could postulate that the treatment use should demonstrate a higher risk of SIP over prophylactic use but that was not the trend. There are several reasons why the inverse could be possible. The first is based on the total numbers exposed to indomethacin. By exposing indomethacin to only those infants who require it for treatment, the total number of SIPs in relation to indomethacin would be reduced. Another reason for prophylactic use to confer a higher risk of SIP could be in relation to timing. SIP occurs most commonly in the first 3 days of life which is the same window when indomethacin prophylaxis is administered, while treatment use typically starts later than the first days of life (1). It is possible that giving indomethacin in this early window might add insurmountable strain in an already high-risk period. To parse out those finer details, knowing the precise timing of medication administration to SIP would be required.

There were two retrospective cohort studies that looked at feeding and indomethacin. From their comparisons, it was evident that feeding premature infants and taking indomethacin had a higher risk of SIP, NEC, and death before discharge compared to those who did not take indomethacin. When infants were not feeding, there was no evidence of an increased risk of SIP, NEC, or death before discharge. These results are in keeping with the non-selective action of indomethacin and subsequent decrease in mesenteric blood flow (11). When blood flow to the gut is reduced, challenging it with feeds may add additional stress. The articles did not explore advancing feeds while taking indomethacin, but caution should be taken.

There has been a slow shift away from indomethacin RCTs with only one article (Davidson, 2021) using indomethacin since 2012 (59). Whether this is due to a lack of equipoise or due to decreased use is uncertain. A 2023 Cochrane review showed that while indomethacin reduces severe IVH, it does not affect the composite outcome of moderate/severe neurodevelopmental disability (101). Additionally, with prophylactic low-dose hydrocortisone protocols now coming into focus for BPD reduction, recommendations are to not use indomethacin and steroids concurrently (102). For PDA closure indomethacin and ibuprofen have similar efficacy, with ibuprofen having a more selective mechanism (47). Cumulatively, the evidence demonstrates that the targeted population for indomethacin is perhaps narrower than first envisioned.

As PDA management evolved, ibuprofen has proven to be a contender. With respect to ibuprofen exposure and the risk of SIP, the major clinical questions include the following: what is the general risk of SIP, is it riskier than indomethacin, and does the administration route alter the risk profile? Our meta-analysis revealed that there was no difference in risk of developing SIP when comparing ibuprofen to no medication. When comparing it head-to-head with indomethacin, both study designs (RCTs and cohort) did not reach significance; however, the results trended toward less risk of SIP in those taking ibuprofen vs. those taking indomethacin. The same trends held true for the secondary outcomes of NEC and oliguria. In general, this is in keeping with the notion that while both indomethacin and ibuprofen are NSAIDs, the side effects profile for ibuprofen is milder. For PDA management oral ibuprofen has been shown to be more efficacious than intravenous (51). Making the next logical question, does route of ibuprofen administration change the risk of SIP. Based on our analysis there was no difference in the pooled proportion of SIP in premature infants taking ibuprofen via oral or IV routes. Overall, the meta-analysis trends showed ibuprofen to confer less risk of SIP than indomethacin, and there was no difference in the proportion of SIP based on administration route.

In PDA treatment, there are less trials looking at acetaminophen which was reinforced in this review. There were three RCT studies that explored acetaminophen compared to other medications. Of the 107 patients exposed to acetaminophen, there were 3 cases of SIP (Supplementary Data Sheet 3). Given the low sample size, this is not enough to draw any conclusions. Acetaminophen is the medication of choice when there are contraindications to NSAIDs; thus, looking only at retrospective cohort data would introduce selection bias. Until it becomes more prominent the comparative evidence between acetaminophen and NSAIDs, characterizing SIP will be insufficient.

Another way to map the clinical practice landscape is to look at the trends over time. This can be done on a large scale exploring the incidence of SIP and medication exposure over time or alternatively by focusing on a shorter period to reduce potential background noise. Figure 2 depicts the incidence (as a percentage) of SIP over time, showing first the indomethacin era followed by ibuprofen. In keeping with the trends of the meta-analysis, ibuprofen has a lower percentage of SIP than indomethacin. Interestingly, both were decreasing until the last few years when it subjectively started and continued to rise. One factor that may have contributed to the rise in SIP is the lower age of viability. The more immature the infant, the more immature the gastrointestinal function and motility, thus increasing the risk of SIP. One way to explore this postulation would be to look at the incidence of SIP by gestational age. Additionally, diagnostic capabilities are improving, allowing more cases of SIP to be detected. The combination of these factors could be adding to the increase in SIP. This review did not limit medications based on their therapeutic indication and used a wide publication time frame. To limit this scope, we completed a subgroup analysis looking at just the last decade of ibuprofen RCTs. The expectation was decreased heterogeneity. However, the pooled proportion of SIP within the last decade compared to the prior was the same, with similar heterogeneity. This suggests that despite a changing landscape the risk of developing SIP with ibuprofen exposure has remained unchanged. This, combined with the increasing incidence of SIP, further supports the idea that earlier age of viability and better detection are contributing to the increase in the incidence of SIP.

This systematic review is the first step in comprehensively gathering what is known to date about common medications and SIP in premature neonates. In laying that foundation, it was important to keep the scope wide to analyze the general trends. From here, one can narrow the scope, via time, definition, which medication, the medication administration route, or geography to flesh out more granular details.

### Limitations

The results of our analyses must be viewed with an understanding of the limitations. The diversity of the content resulted in high heterogeneity which had several contributing factors including numerous research questions, geographical care differences, and early SIP nomenclature concerns. Articles were not selected based on SIP being the primary outcome; rather, regardless of the research question, the data were probed to determine medication exposures and the occurrence of SIP. Earlier articles looked to find commonalities among cohorts, while others focused on PDA treatment with SIP as an adverse outcome.

One of the first hurdles in paper accruement was crafting a definition of SIP that predated the now-accepted version. In numerous formative papers, authors would include SIP under a general NEC umbrella. In the included list of RCT articles, 19 of the 25 were from after the 2002 official NICHD definition of SIP (4). It is possible that while the attempt was to provide an in-depth review of SIP solely, many cases have not been captured due to nomenclature issues as the neonatal field was actively expanding and evolving. This was most evident when discussing SIP but was also true for secondary outcomes. Having consistent reporting of definitions and standards would improve what data can be extracted for future meta-analysis comparisons.

Another paper accruement issue was that the many papers identified used NSAIDs for PDA management. Unless the abstract mentioned SIP it would not have been detected in the search strategy. Possible next steps could entail searching for all papers involving PDA management with NSAIDs and then excluding articles during full-text screen that did not report SIP.

An additional contributor to the high heterogeneity could be differences in global practice styles. To increase catchment, searches were worldwide with no language restrictions. A subgroup analysis based on geography was not conducted. Of note, 36 of the 65 articles included arose from North America. With the incidence of SIP increasing, there is a need for focused initiatives to decrease risk where possible, and exploring regionalized themes will be of future clinical relevance.

Understanding the timing of medication exposure to the presentation of SIP was a limiting factor. Studies recorded medication exposure and SIP in a binary fashion. However, to discern the true relationship, knowing the timing of medication exposure to the onset of SIP is crucial. The closest to this was the comparison of prophylaxis to the treatment use of indomethacin. Understating the temporal relationship could help identify a potential high-risk timing window.

## Conclusions

This is the first systematic review exploring the impact of common NSAID medications on the risk of neonatal SIP over the last four decades. While cases of SIP with indomethacin or ibuprofen exposure were declining, it is now on the rise. We documented the challenges of nomenclature as SIP evolved into a unique entity. Indomethacin or ibuprofen alone vs. no medication does not increase the risk of SIP. However, treatment use trended toward less risk of SIP than prophylactic use. When exploring the relationship of indomethacin and feeds, infants not fed had less risk of SIP. Indomethacin should be restricted to a narrower subpopulation of premature infants with the implementation of stricter criteria. Broadly, ibuprofen was gentler than indomethacin, and the route of ibuprofen administration does not alter the risk of SIP.

Moving forward, the field needs nomenclature unification and timing of medication exposure to the onset of SIP to tease out the details of gastrointestinal pathology in premature neonates. As more data become available, limiting the search field time-wise, making geographic comparisons, and continued use of strict outcome definitions could lend itself to a stronger consensus for classification. This will lead to a transparent interpretation of the data and will delineate further details on the evolving relationship between commonly used medications and neonatal SIP.

## Data Availability

The original contributions presented in the study are included in the article/Supplementary Material, further inquiries can be directed to the corresponding author.
