# Supplementary material for: Use of NSAIDs and acetaminophen and risk of spontaneous intestinal perforations in premature infants: a systematic review and meta-analysis
Source: Front Pediatr. 2024 Nov 22;12:1450121. doi: 10.3389/fped.2024.1450121 (PMC11620902; doi:10.3389/fped.2024.1450121)
Supplement: Supplementary file 1 [file Presentation1.zip › Supplementary Data Sheet 3.pdf]

Supplementary Material

Association between use of indomethacin, ibuprofen and acetaminophen with spontaneous intestinal perforation (SIP) in preterm infants

Objective of this SR
The primary objective of this systematic review is to investigate the incidence of SIP in premature infants who have been exposed to either Indomethacin, Iuprofen, or Acetaminophen.

In this meta-analysis we excluded case series and case reports.

Note: In the following analyses, we will not report pooled estimates if the heterogeneity $I^2 > 75\%$.

Primary Outcome: Spontaneous Intestinal perforation meta-analyses.

1. What is the risk of SIP in premature infants who receive Indomethacin medication compared to no medication?

Figure 1-A: Forest plot of Indomethacin (Treatment or Prophylaxis) versus no medication for RCTs only.

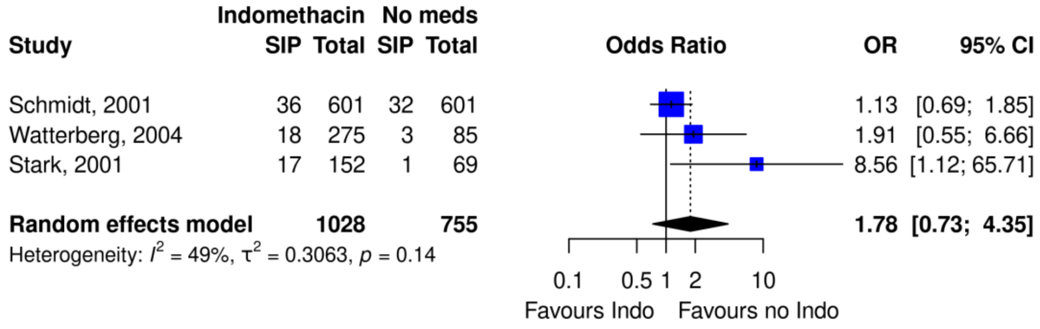

The RCT pooled estimate shows no evidence of a difference in the risk of SIP between Indomethacin and no Indomethacin group, the CI overlaps 1.

Figure 1-B: Forest plot of Indomethacin (Treatment or Prophylaxis) versus no medication. Cohort studies only.

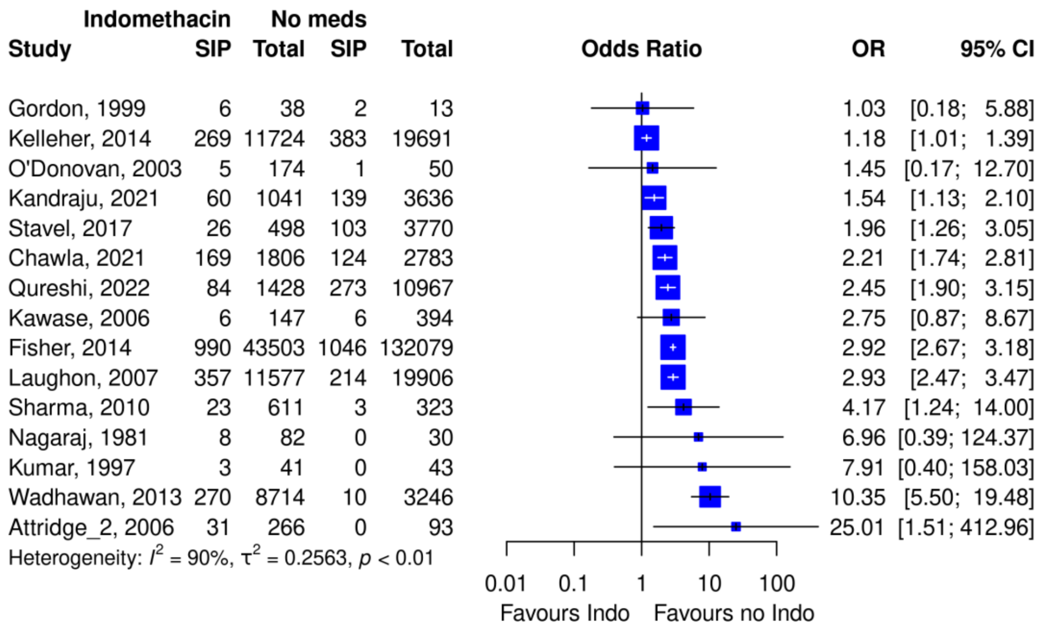

There is high heterogeneity therefore we didn't pool the estimate.

Figure 1-C: Forest plot of Case Control studies: Odds ratio of patients who are taking any Indomethacin (Treatment or prophylaxis) in cases of SIP compared to controls.

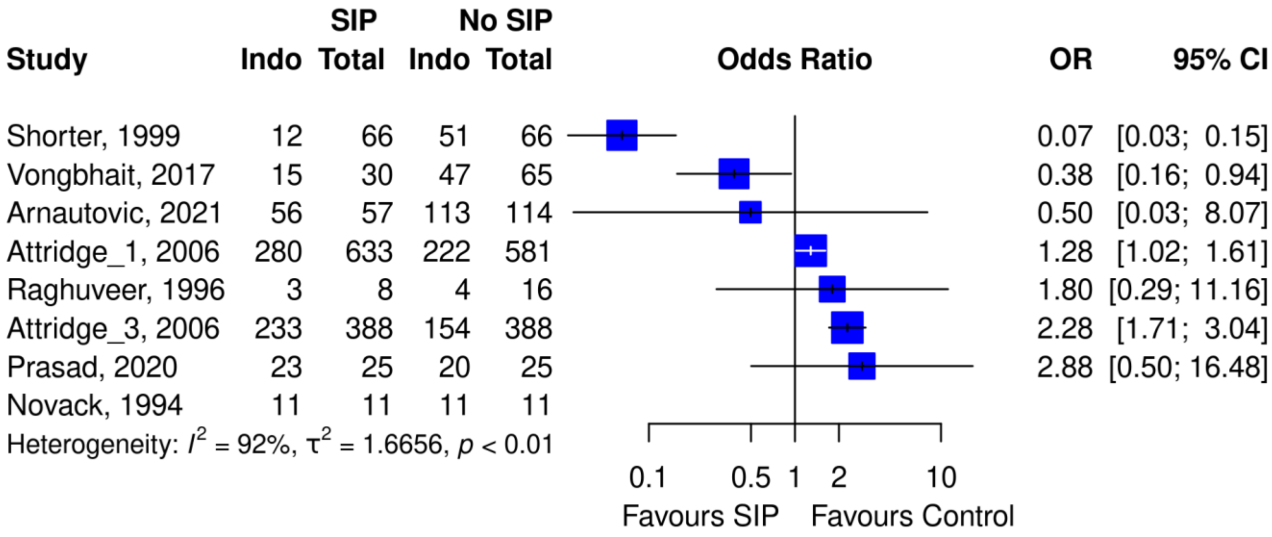

Pooled OR was not reported due to high heterogeneity.

2. What is the risk of SIP in premature infants who receive Ibuprofen medication compared to no medication?

Figure 2-A: Forest plot showing the risk of SIP in premature infants taking Ibuprofen versus no medication (All studies are RCTs).

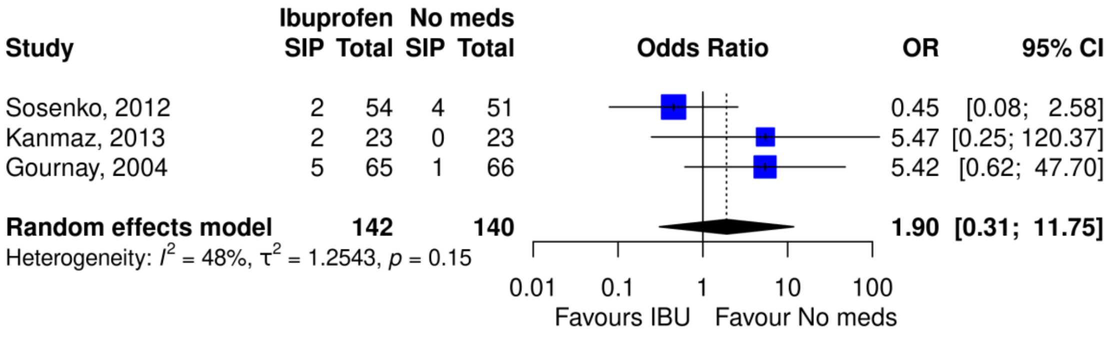

There is no evidence that there is an increased in the odds of SIP in infants taking Ibuprofen compared to no medication.

Figure 2-B: There is one Case control study of cases SIP in infants taking Ibuprofen compared to controls. (Figure for illustration only).

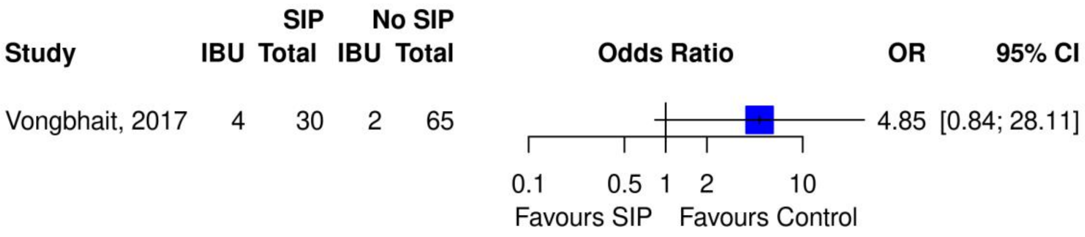

3. What is the risk of SIP in premature infants who receive prophylaxis versus treatment with Indomethacin?

Figure 3: Forest plot showing the risk of SIP in premature infants taking Indomethacin as a treatment vs prophylaxis. (All studies are cohort studies).

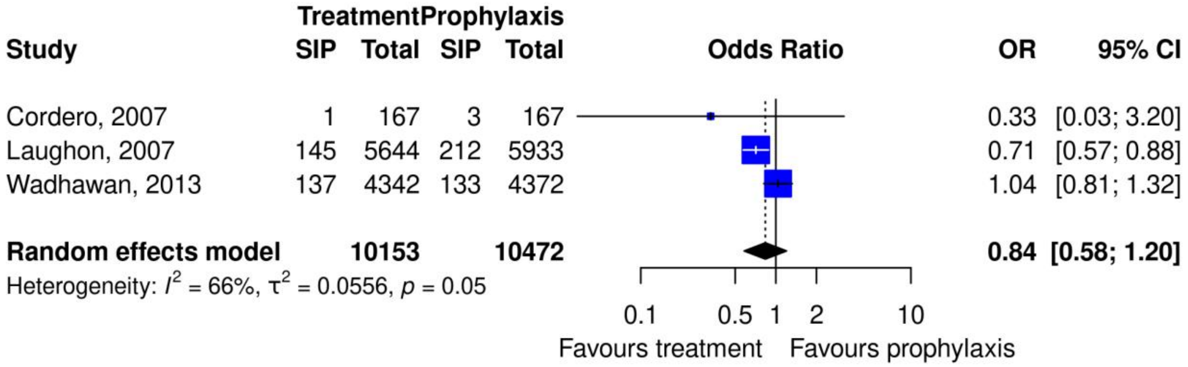

The 2 studies with the higher sample size have conflicting results, as one had an evidence of a lower risk of Indo treatment compared to prophylaxis, while the other study had no significant results with a borderline results, therefore our pooled estimate is borderline and not certain and indicates there is a lower risk of SIP with Indo treatment vs Indo prophylaxis.

We have a new study Zozaya, 2021, a cohort study, one group is Indo_prophylaxis vs Indo/ibu treatment. We did not include it, as it is not a pure comparison.

4. What is the risk of SIP in premature infants who receive one type of medication compared to another?

Figure 4-A: Forest plot for the risk of SIP in infants receiving Indomethacin vs Ibuprofen.

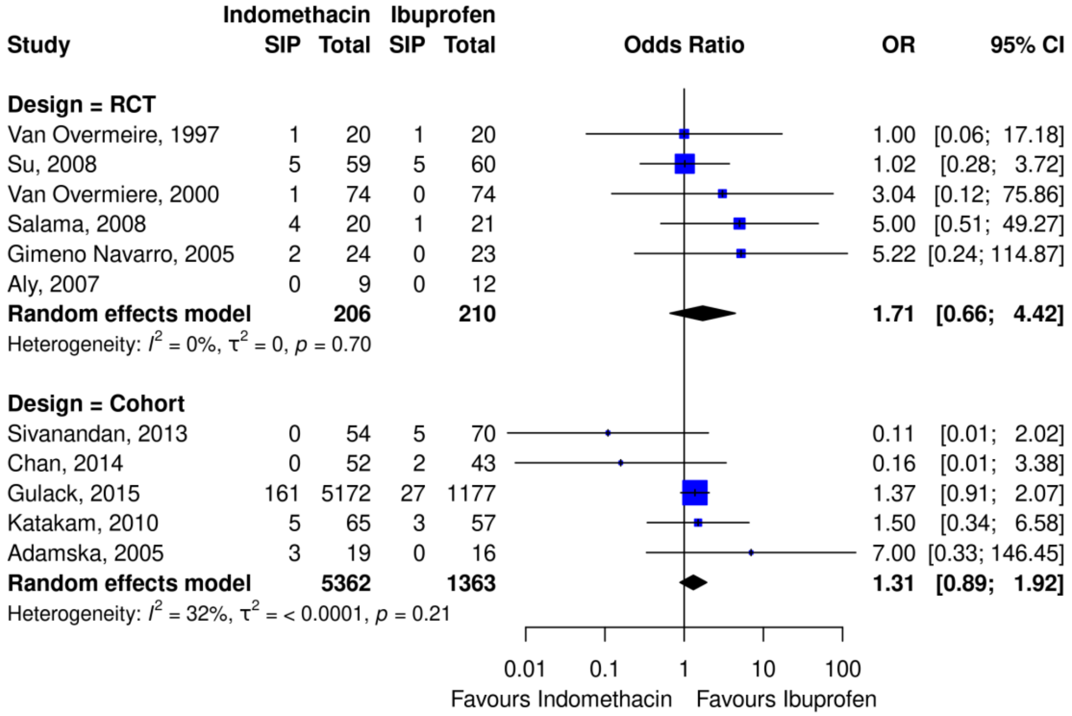

For both study designs (RCTs and Cohort), although it doesn't reach the predetermined margin of significance, however, the results indicate less risk of SIP in those taking Ibuprofen vs Indomethacin, although it didn't reach statistical significance threshold.

Figure 4-B: Forest plot for the risk of SIP in premature infants receiving Acetaminophen vs Indomethacin or Acetaminophen vs Ibuprofen. (for illustration only)

There was a study by Hochwald, 2018 but we removed it, as it was not a pure comparison (Aceta+IBU vs IBU alone).
Also, there was Luecke, 2017: is Aceta (pre post treatment), so not included.

There were not enough studies in both groups so this figure for illustration only.

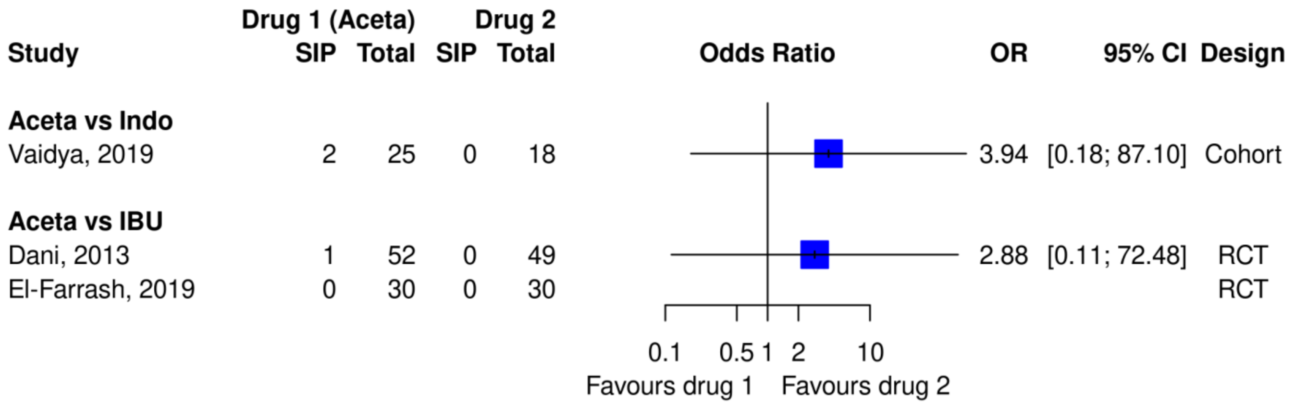

5. Prevalence of SIP in patients taking the medications (analysis of one arm or no control group).

Figure 5: Pooled prevalence of SIP in premature infants taking Indomethacin (any type).

5-A) RCT studies

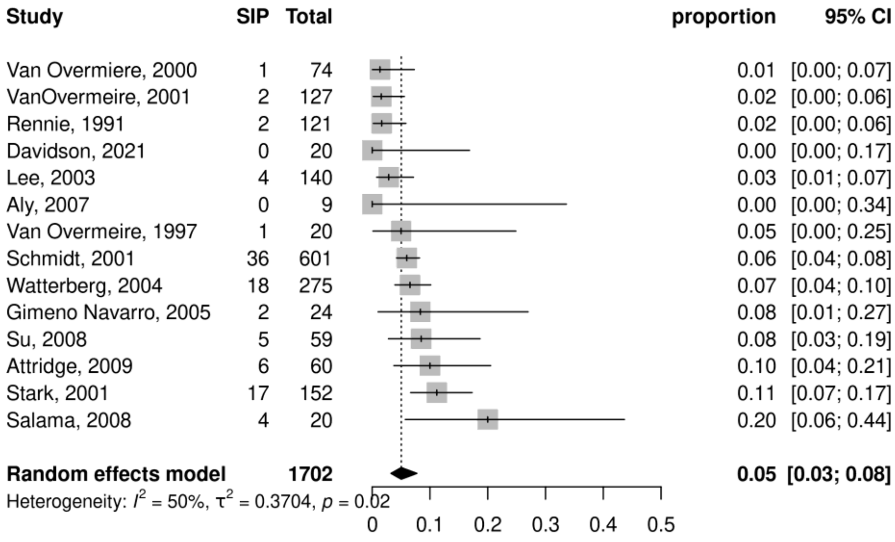

5-B) Cohort studies

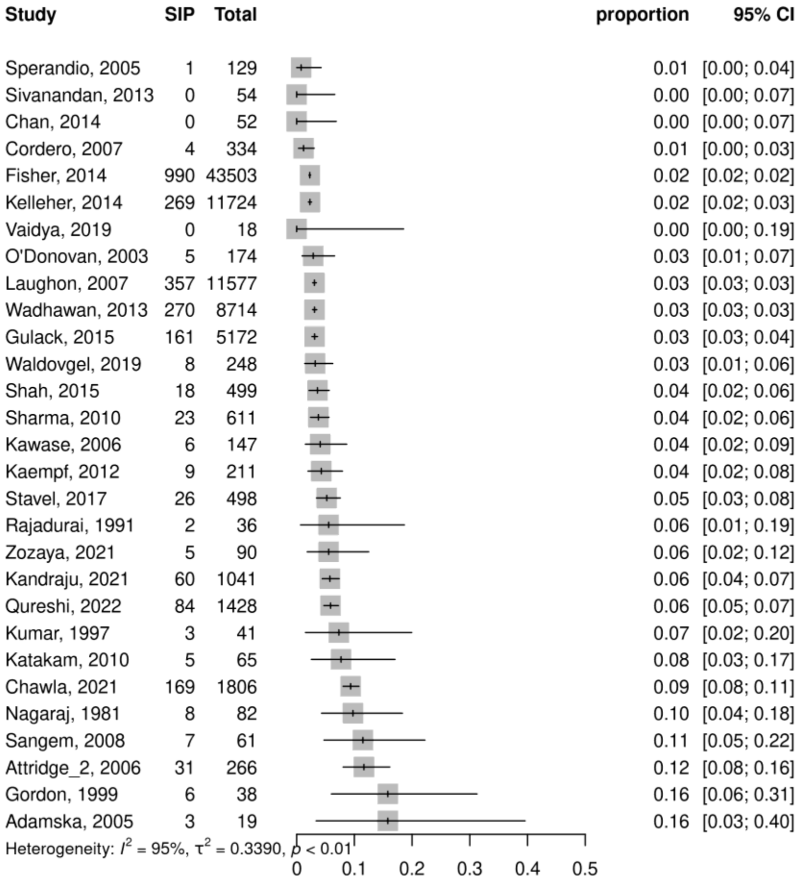

We didn't pool the estimate since the heterogeneity is high.

Figure 6: Pooled prevalence of SIP in premature infants taking Ibuprofen (any route).

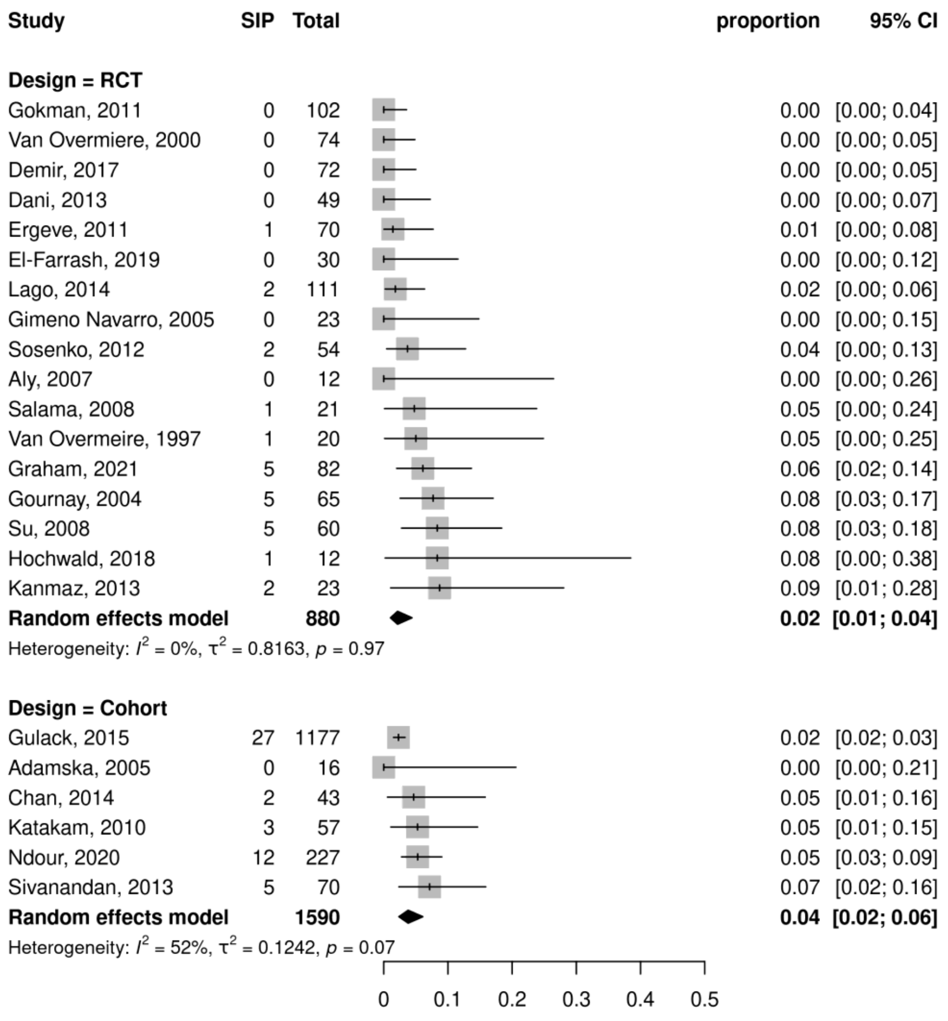

Figure 7: Pooled prevalence of SIP in premature infants taking Acetaminophen.

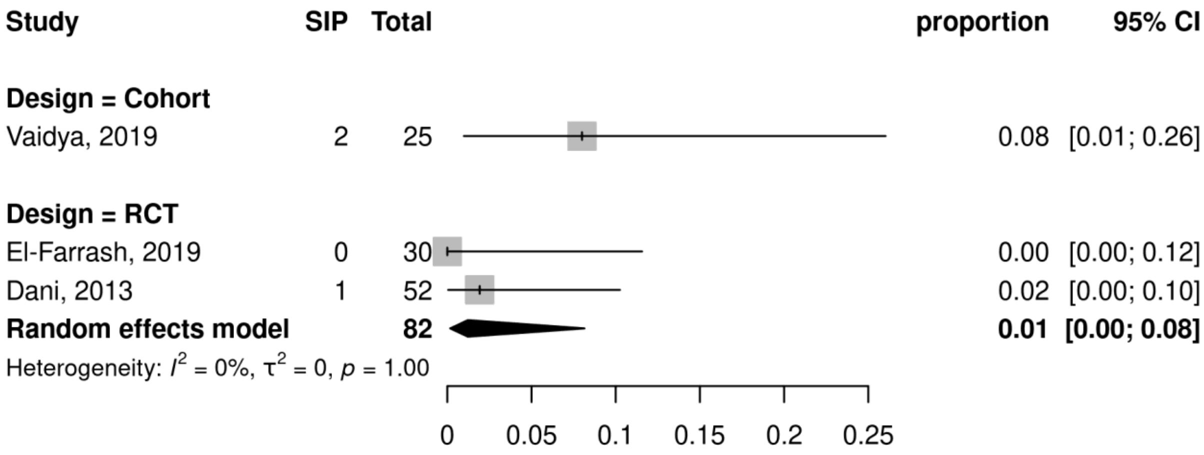

6. Are there differences in the risk of SIP with Indomethacin regarding feeding regimens.

Figure 8: Forest plot for the risk of SIP in premature infants taking Indomethacin vs no medication stratified by feeding regimens.

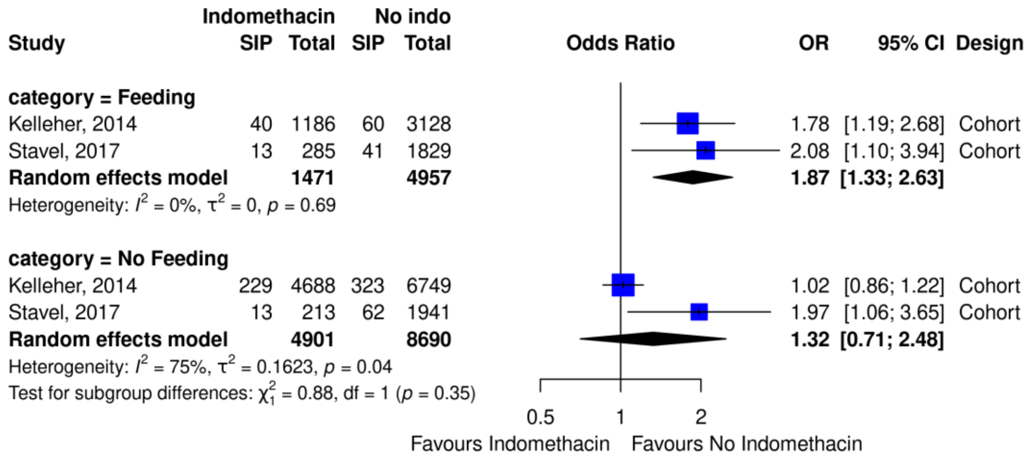

It is evident that premature infants on assisted feeding, and taking Indomethacin have higher risk of SIP compared to those who didn't take Indomethacin.

For those on no feeding, there is no evidence that there is an increased risk of SIP in infants taking Indomethacin compared to not.

We tested for subgroup difference and there is no difference between the 2 feeding categories.

7. (new analysis added): The prevalence of SIP in premature infants taking Ibuprofen stratified by the route (IV vs Oral).

Figure 9-A: Forest plot for the risk of SIP in premature infants taking Ibuprofen stratifiedy by route.(this figure for illustration just to show that there is a study with rectal route). All studies have indirect comparison.

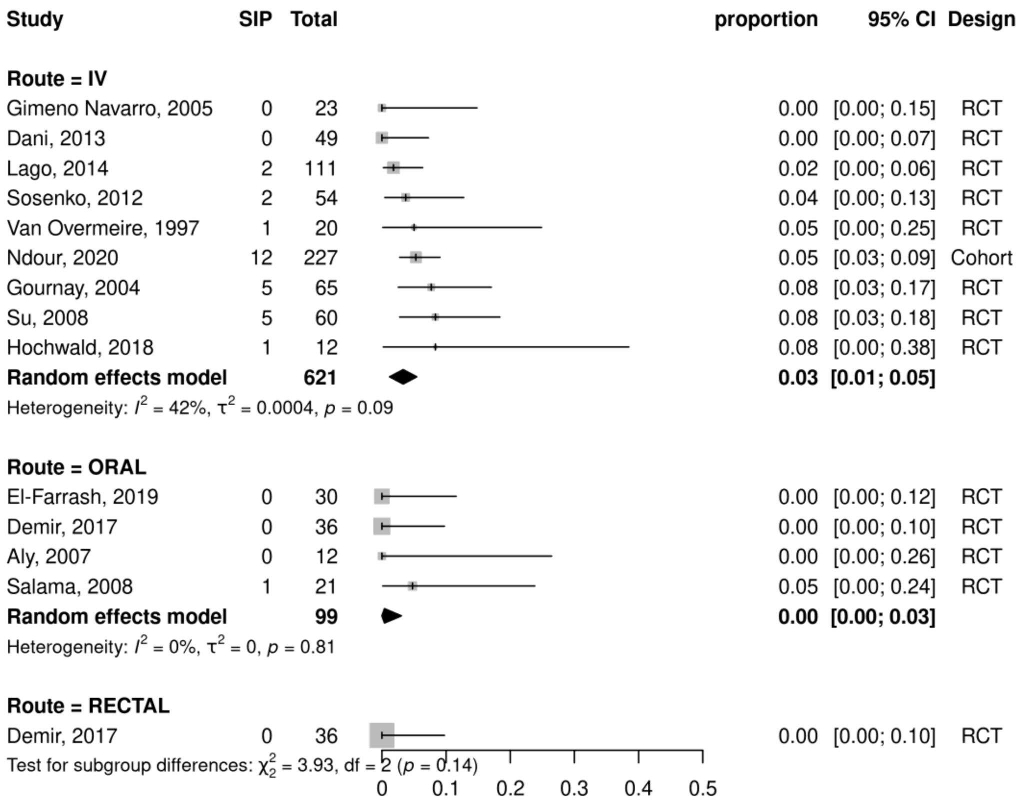

Figure 9-B: Forest plot for the pooled proportion of SIP in premature infants taking Ibuprofen stratified by route (IV vs Oral).

We have one cohort study (Ndour, 2020) so we excluded it since all the other studies are RCTs.

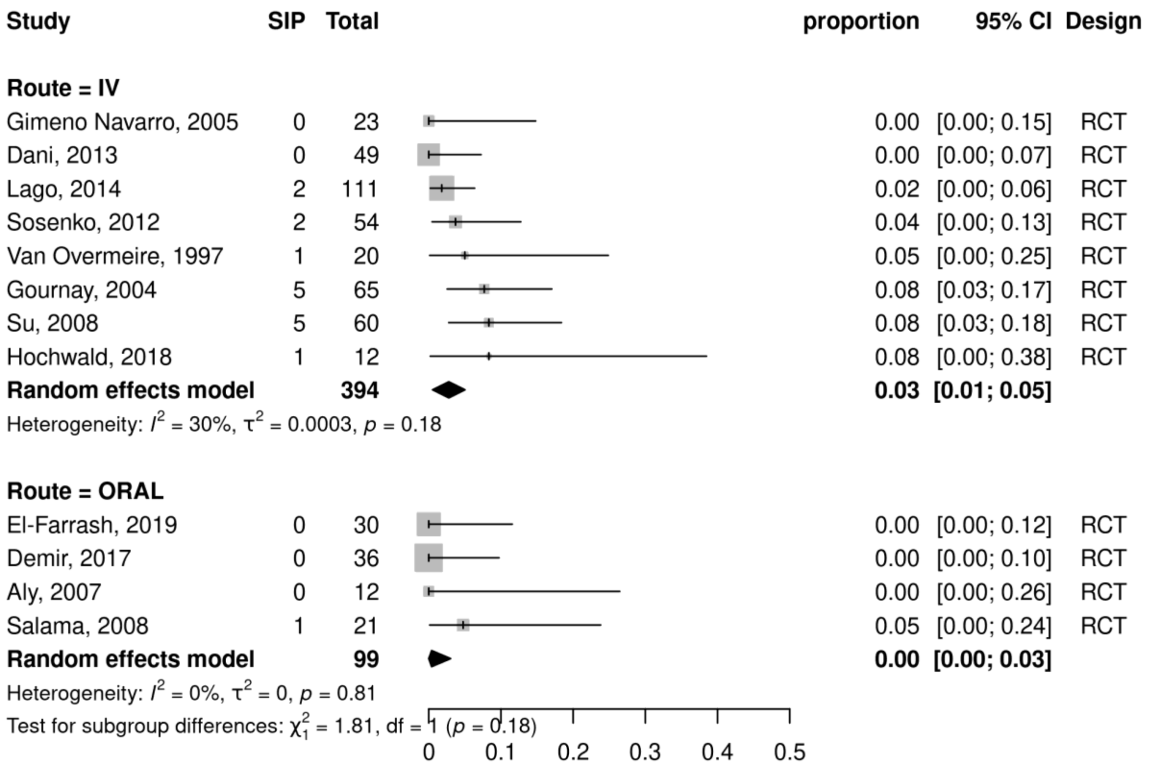

This forest plot shows that there is no evidence of a difference in pooled proportion of SIP in premature infants who are taking Ibuprofen by IV compared to those who are taking it orally, p =0.18

Figure 9-C: Forest plot for the risk of SIP in premature infants taking Ibuprofen- comparative studies of Oral vs IV.

We have 2 comparative studies, but the second one has 0% so the pooled proportion was not estimated and this figure for illustration only

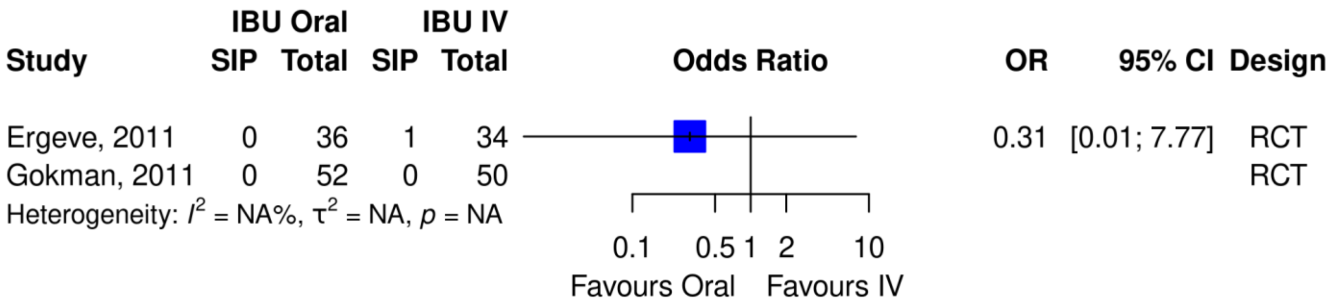

Secondary Outcomes Meta-analyses.

Necrotizing Enterocolitis (NEC) Analyses

NEC Risk for Indomethacin vs control (no Indomethacin)

Figure 10-A: Forest plot for the risk of NEC in premature infants taking Indomethacin vs no medication

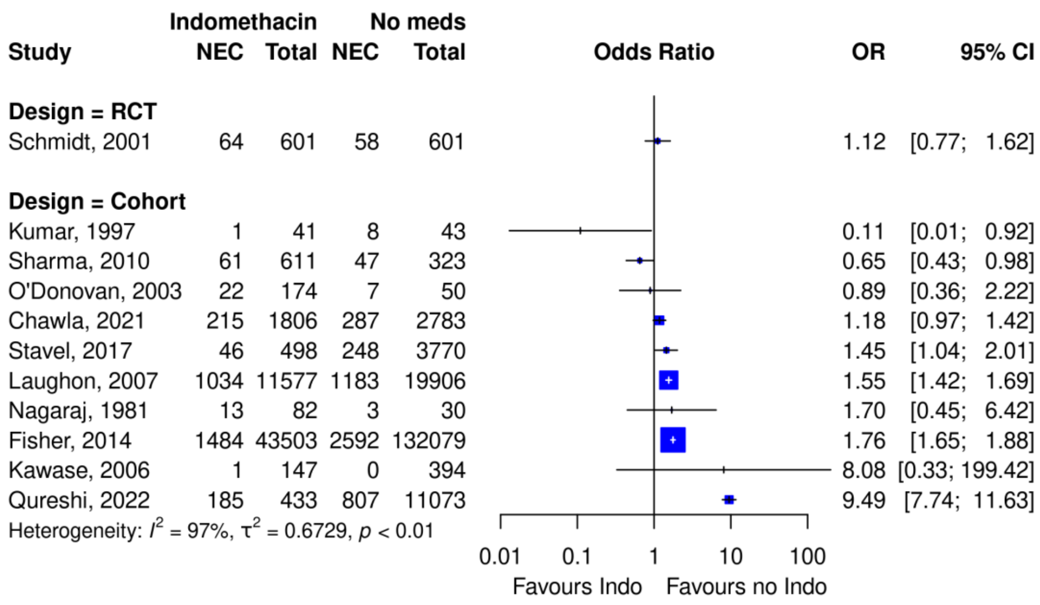

We didn't report the pooled estimate because of the high heterogeneity; I^2= 97%.

Figure 10-B: Odds ratio of patients who are taking any Indomethacin (Treatment or prophylaxis) in cases of NEC compared to controls. There is one study so figure for illustration only.

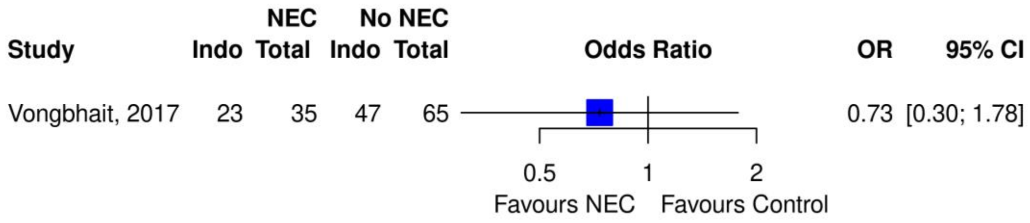

Risk of NEC in premature infants who receive Ibuprofen medication compared to no medication.

Figure 11-A: Forest plot showing the risk of NEC in premature infants taking Ibuprofen versus no medication (there is only one study, so this is not a pooled estimate, the plot is for illustration only)

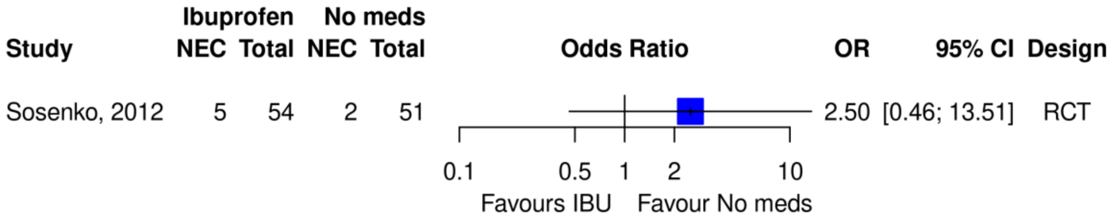

Figure 11-B: there is one Case control study of cases NEC in infants taking Ibuprofen compared to controls. (figure for illustration only).

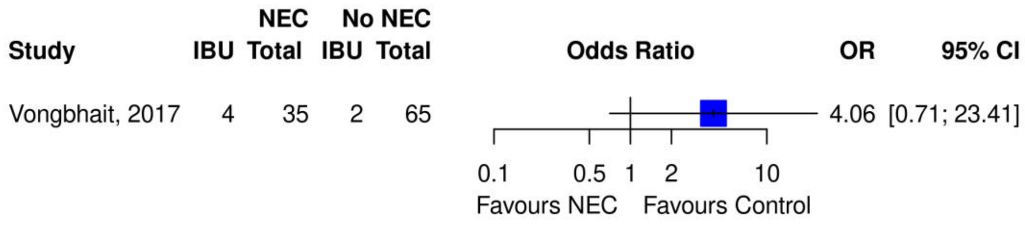

NEC: Indomethacin as a treatment vs prophylaxis.

Figure 12: Forest plot showing the risk of NEC in premature infants taking Indomethacin as a treatment vs prophylaxis.

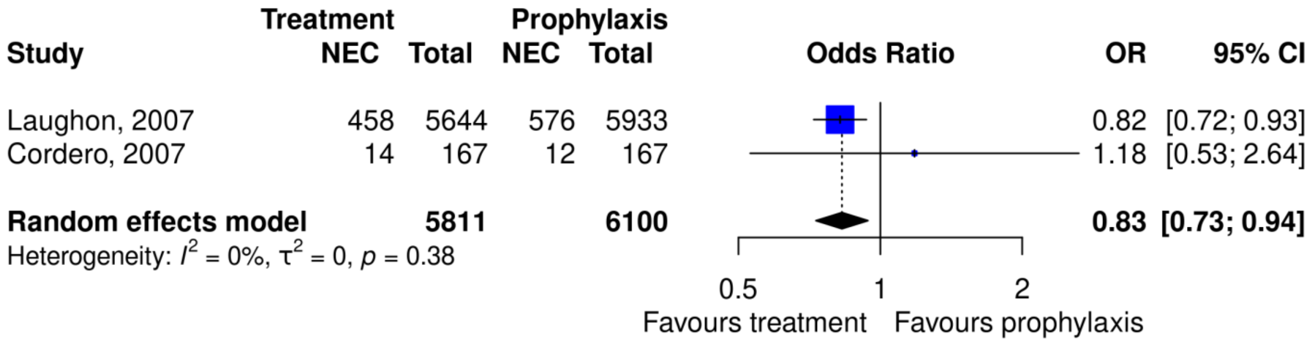

The pooled estimate shows an evidence of a lower risk of NEC in infants taking Indomethacin as a treatment vs as a prophylaxis.

There is one case control study that had Indomethacin Treatment vs Prophylaxis, the figure below for illustration and not a pooled analysis

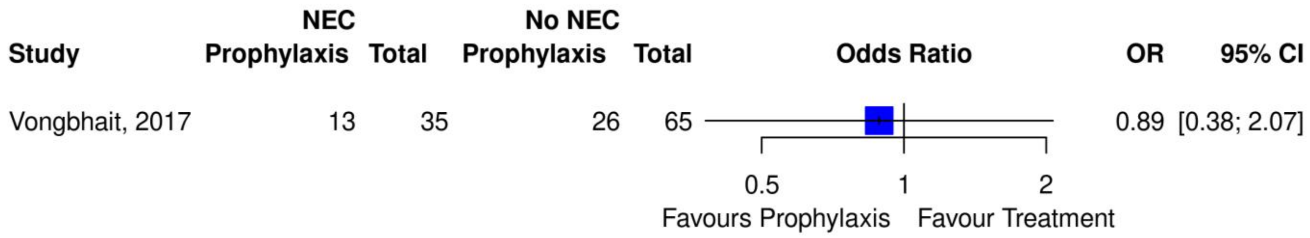

NEC: Risk of NEC in studies of Medications vs other Medications

Figure 13: Forest plot showing the risk of NEC in premature infants taking Indomethacin vs Ibuprofen

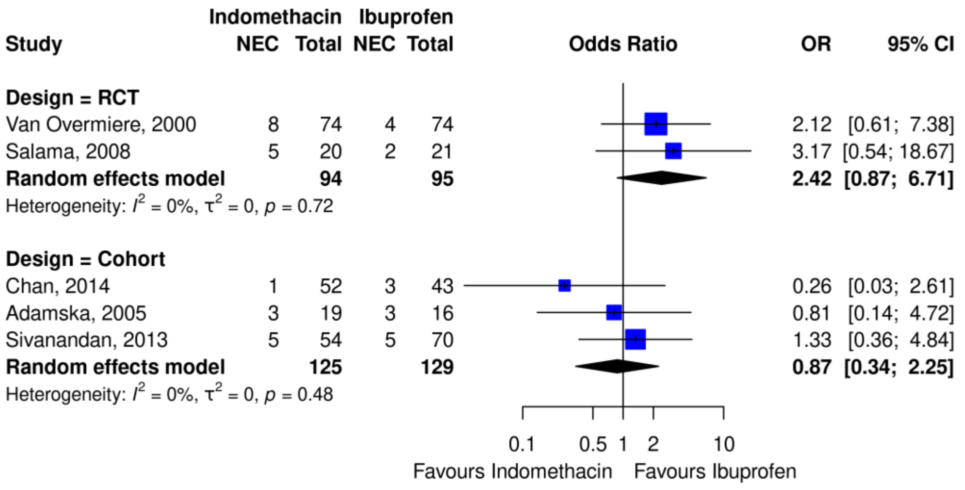

In RCT and Cohort studies, there is no evidence of a difference in the risk of SIP in patients taking Indomethacin compared to Ibuprofen. The 95% CI is wide and crossing one.

There is one case control study that has a group with Indomethacin and a group with IBU, the figure below is for illustration only and not part of the meta-analysis.

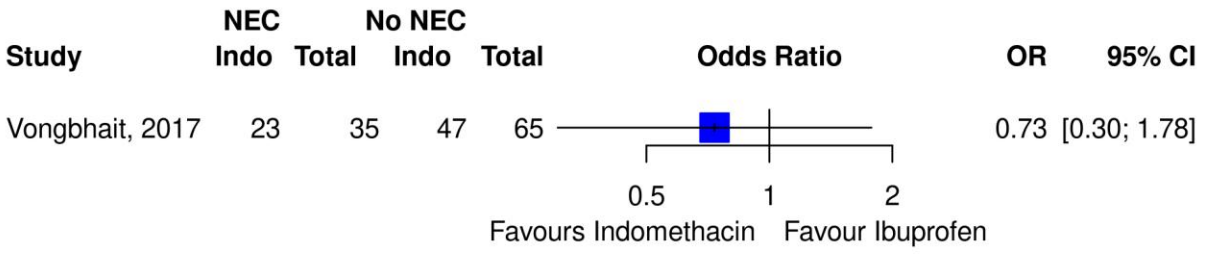

NEC: Acetaminophen vs other medication

There is only one study by Hochwald of Acetaminophen + IBU vs IBU alone, and we recommend not to include this study

Prevalence of NEC in patients taking medications (analysis of one arm with no control group).

Figure 14-A: Pooled prevalence of NEC in premature infants taking Indomethacin (RCT studies)

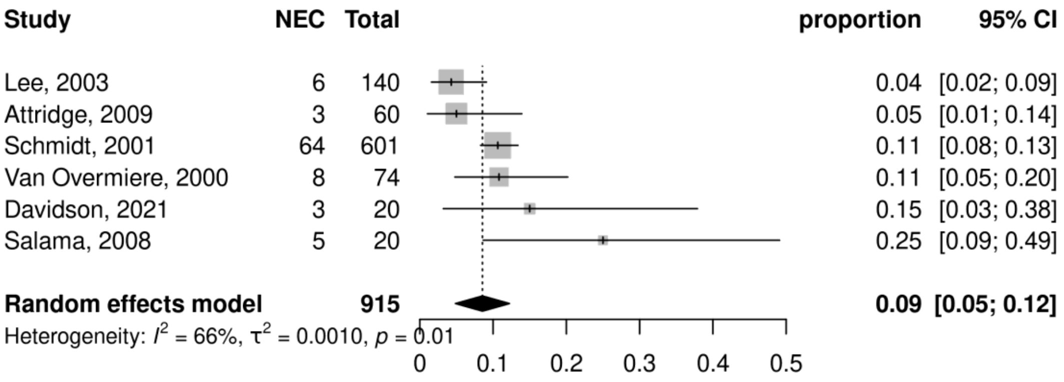

Figure 14-B: Pooled prevalence of NEC in premature infants taking Indomethacin (Cohort studies).

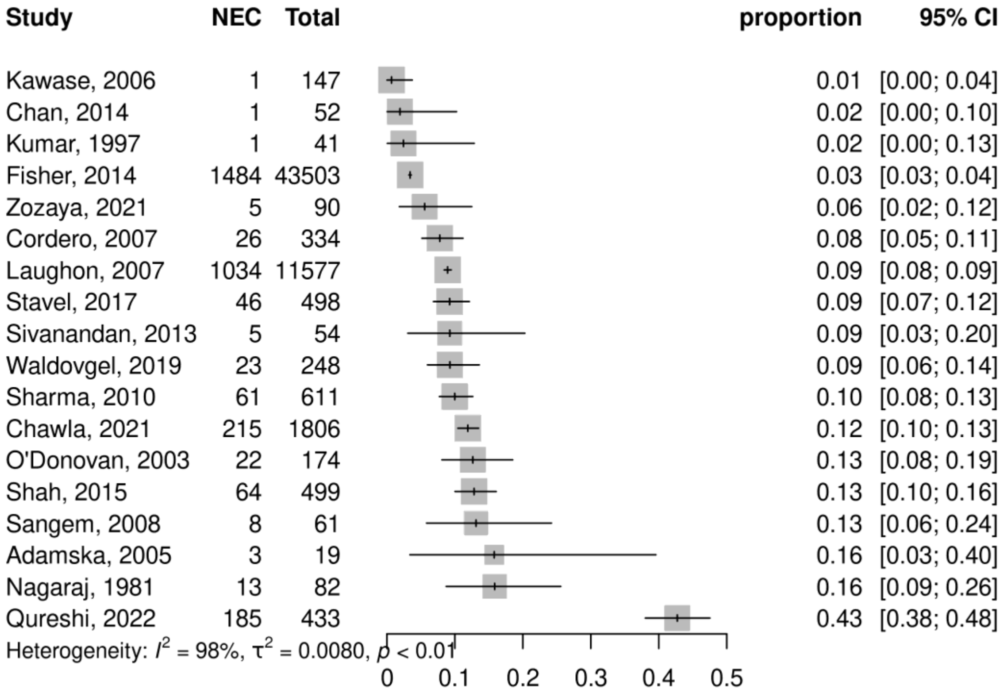

We didn't report the pooled proportion because the heterogeneity was very high.

Figure 15: Pooled prevalence of NEC in premature infants taking Ibuprofen.

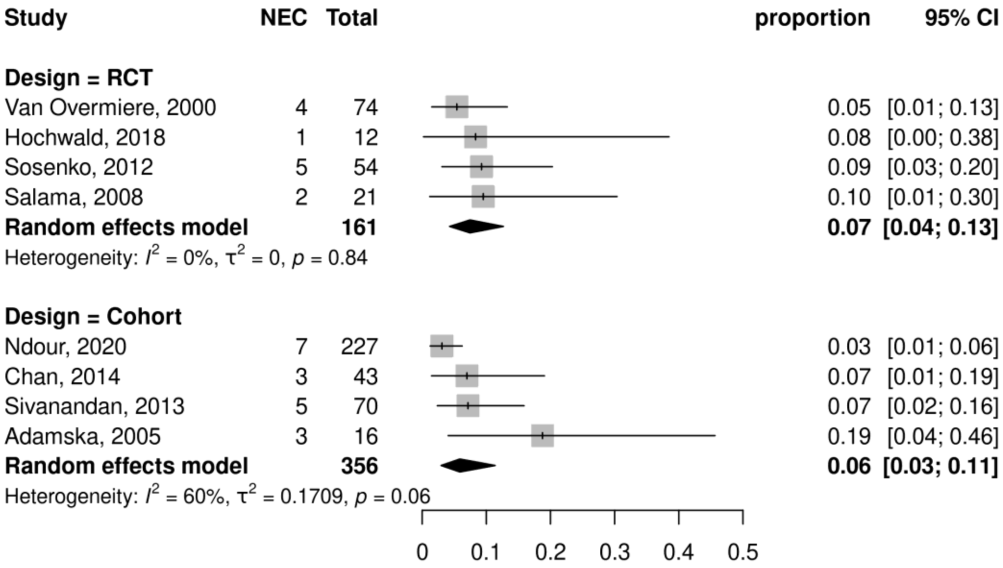

Prevalence of NEC in premature infants taking Acetaminophen.

There is only one study of Acetaminophen with Ibuprofen by Hochwald.

Are there differences in the risk of NEC in premature infants taking Indomethacin regarding feeding regimens.

Figure 16: Forest plot for the risk of NEC in premature infants taking Indomethacin vs no medication stratified by feeding regimens.

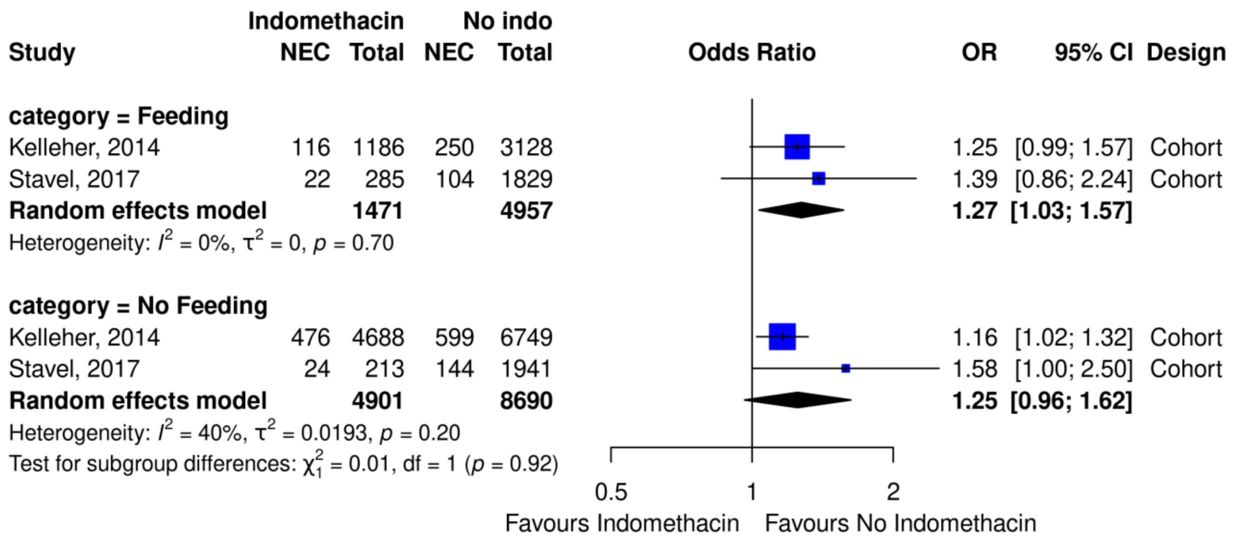

It is evident that premature infants on assisted feeding, and taking Indomethacin have higher risk of NEC compared to those who didn't take Indomethacin.

For those on no feeding there is borderline evidence that there is an increased risk of NEC in infants taking Indomethacin compared to not. There is no difference between the 2 groups.

The prevalence of NEC in premature infants taking Ibuprofen stratified by the route (IV vs Oral).

Figure 17: Forest plot for the risk of NEC in premature infants taking Ibuprofen stratified by route.

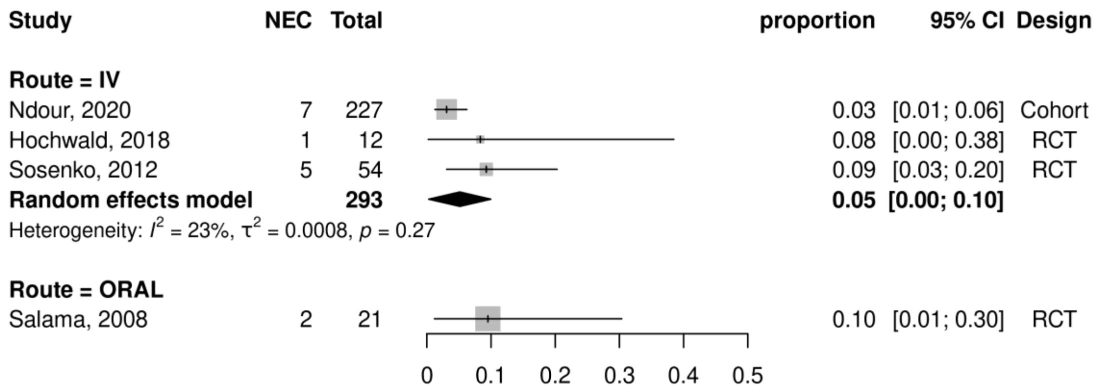

There is only one study in the oral group, so we cannot compare the 2 groups. There are no comparative studies that compared between oral and IV IBU route and have NEC outcome.

Oliguria Analyses

There is only one study (RCT) for the comparison between Indomethacin and control, therefore we cannot pool the estimate.
There are no case control studies that reported oliguria.
There are no studies that had a comparison between Ibuprofen and Placebo and reported oliguria outcome.
There are no studies that compared Indomethacin as a treatment vs prophylaxis and had oliguria outcome.

Figure 18: Forest plot showing the risk of Oliguria in premature infants taking Indomethacin vs Ibuprofen

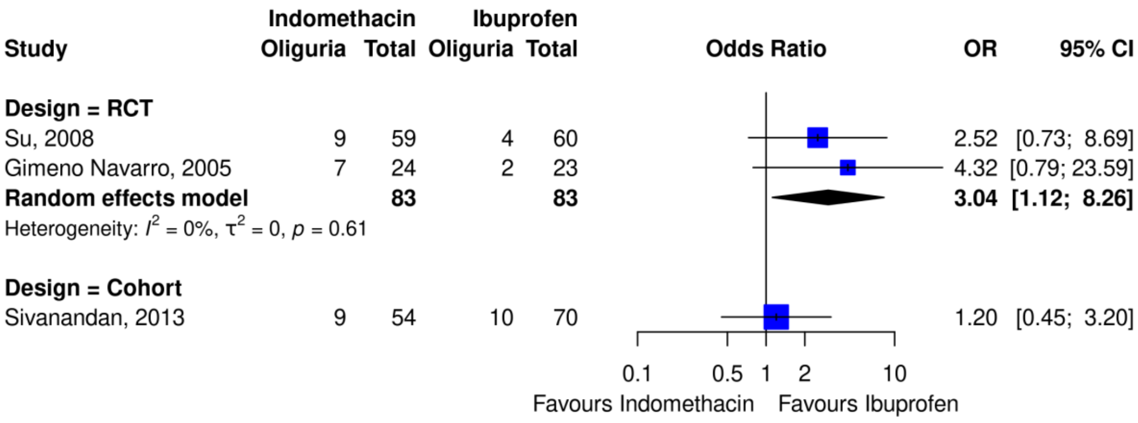

In the pooled estimate of RCT studies, there is evidence that infants taking Indomethacin have higher odds (or risk) of oliguria compared to those taking Ibuprofen.

Figure 19: Forest plot showing the risk of Oliguria in premature infants taking Acetaminophen vs Ibuprofen.

There is one study the figure is for illustration only.

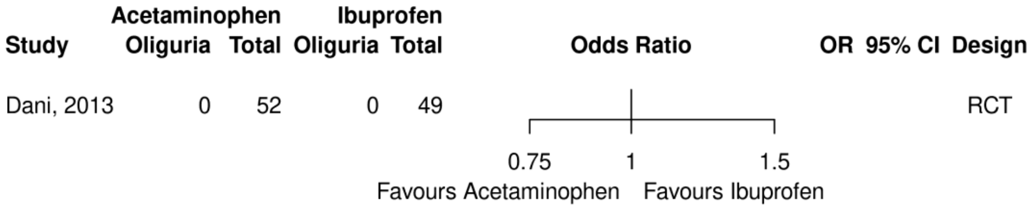
Study
Acetaminophen
Oliguria
Total
Ibuprofen
Oliguria
Total
Odds Ratio
OR 95% CI
Design
Dani, 2013
0
52
0
49
RCT
0.75
1
1.5
Favours Acetaminophen
Favours Ibuprofen

Prevalence of Oliguria in patients taking medications (analysis of one arm with no control group).

Figure 20: Pooled prevalence of Oliguria in premature infants taking Indomethacin (RCT studies)

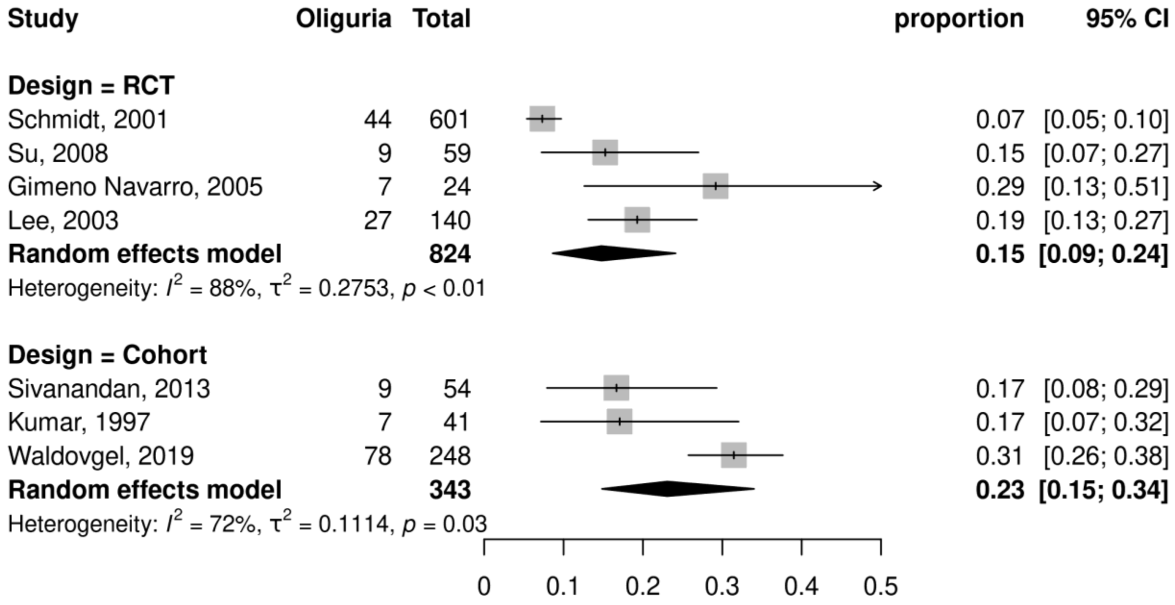

Don’t report the estimate for the RCT as the heterogeneity is high, only report it for the cohort studies.

Figure 21: Pooled prevalence of Oliguria in premature infants taking Ibuprofen

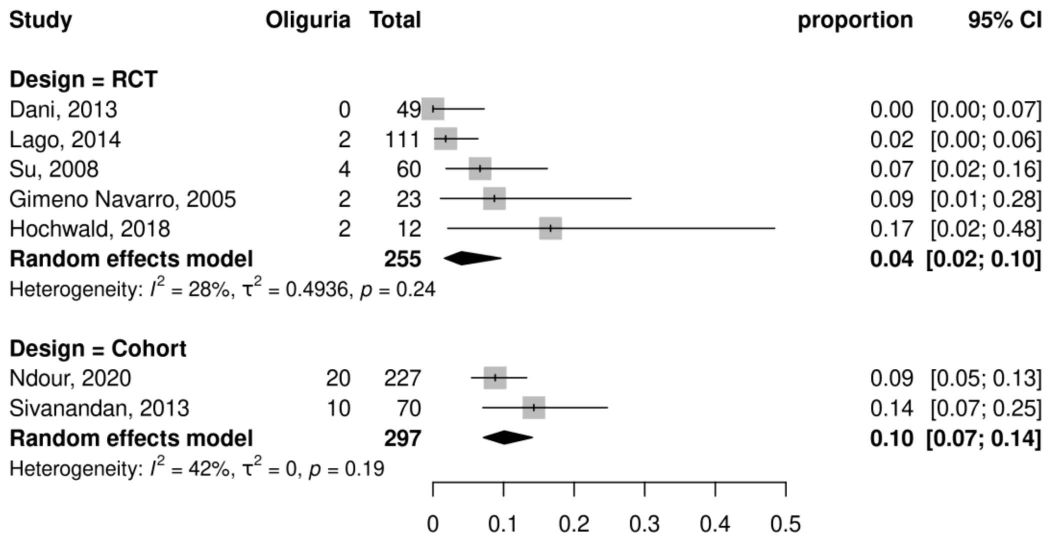

Figure 22: Pooled prevalence of Oliguria in premature infants taking Acetaminophen.

There is one study only, the figure is for illustration only.

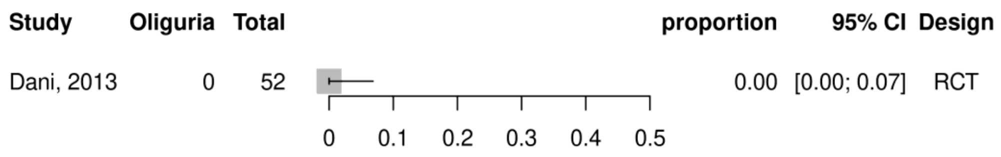

The prevalence of Oliguria in premature infants taking Ibuprofen stratified by the route (IV vs Oral).

All studies that reported oliguria with IBU route were IV route only.

Are there differences in the risk of Oliguria in premature infants taking Indomethacin regarding feeding regimens.

There are no studies related to this comparison reported oliguria as an outcome.

Death before discharge Analyses

Figure 23: Forest plot showing the risk of death before discharge for premature infants taking Indomethacin vs no medication.

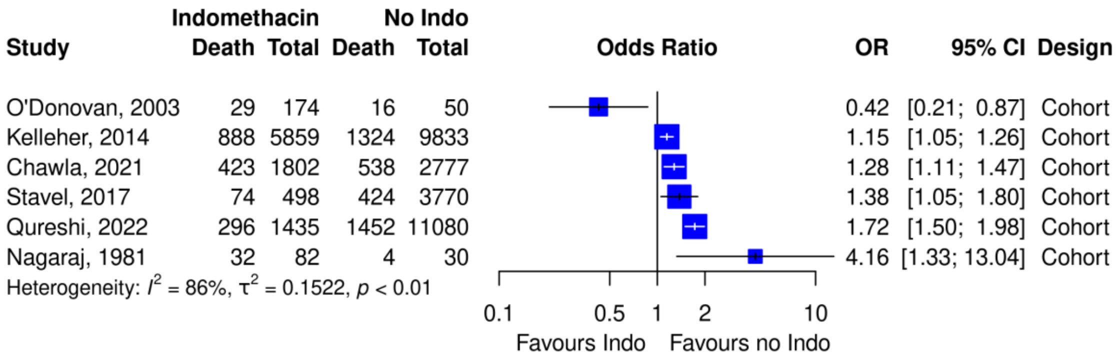

We did not pool the estimate as the heterogeneity is high.

Note: Previous dataset Kumar 1997, and Kandraju, 2021 had data on death before discharge but this is not available in this new dataset

Risk of death before discharge for premature infants taking Ibuprofen vs no medication.

There is only study of IBU vs Control reporting death before discharge.
Previously, there were data for Kanmaz 2013 as well but now it is not

Risk of death before discharge in premature infants taking Indomethacin as Treatment vs Prophylaxis.

There are no studies that have this outcome for these 2 groups, however in previous dataset we had Laughon and Cordero

Risk of death before discharge in studies of Medications vs other Medications

Figure 24: Forest plot showing the risk of death of discharge in premature infants taking Indomethacin vs Ibuprofen

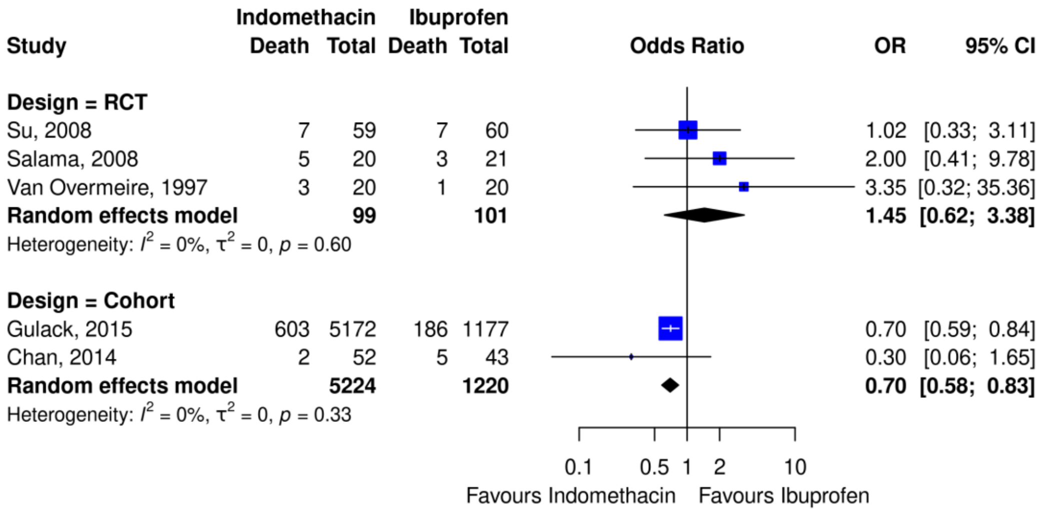

In RCT studies there is no evidence of a difference in the risk of death between infants taking Indomethacin vs Ibuprofen. In cohort studies there was evidence of a lower risk of death before discharge in infants taking Indomethacin vs those taking Ibuprofen.

Please note that in the previous data, there was data for this outcome in Indo vs IBU in 2 studies: Katakam and Sivananden.

Risk of death before discharge in infants taking Acetaminophen vs other medications.

we have no studies for this outcome in this group, but previously El-Farrash study had numbers in this group for this outcome?

Prevalence of death predischarge in patients taking the medications (analysis of one arm or no control group).

Figure 25: Pooled prevalence of death pre-discharge in premature infants taking Indomethacin (any type) for RCT studies only.

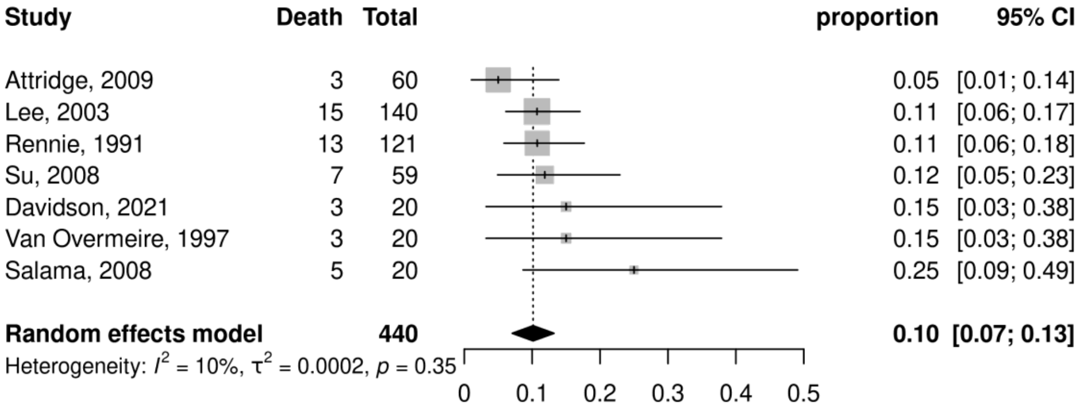

Figure 26: Pooled prevalence of death pre-discharge in premature infants taking Indomethacin (any type) for Cohort studies only.

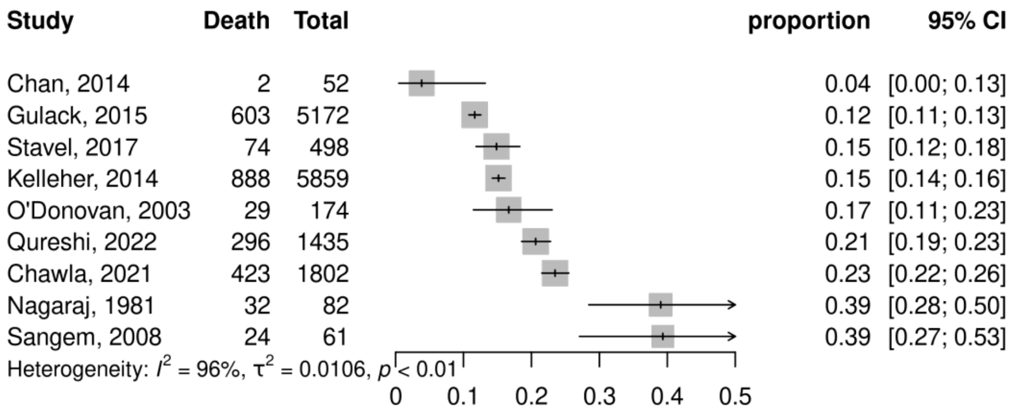

We didn't pool the estimate because of the high heterogeneity.

Figure 27: Pooled prevalence of death pre-discharge in premature infants taking Ibuprofen.

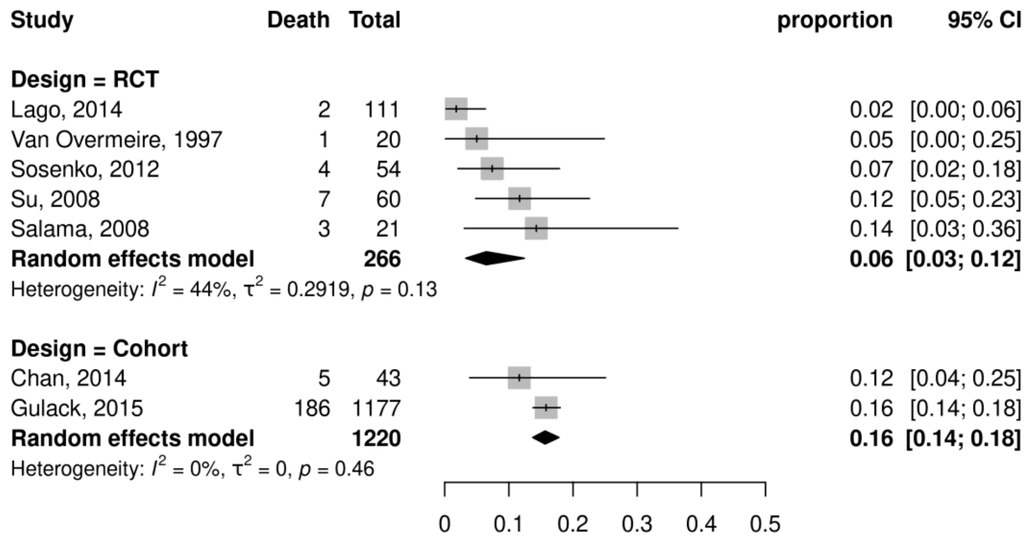

Prevalence of NEC in premature infants taking Acetaminophen

There are no studies for Acetaminophen that reported death before discharge.

Are there differences in the risk of death pre discharge with Indomethacin regarding feeding regimens.

Figure 28: Forest plot for the risk of death pre-discharge in premature infants taking Indomethacin vs no medication stratified by feeding regimens.

Both studies are cohort studies.

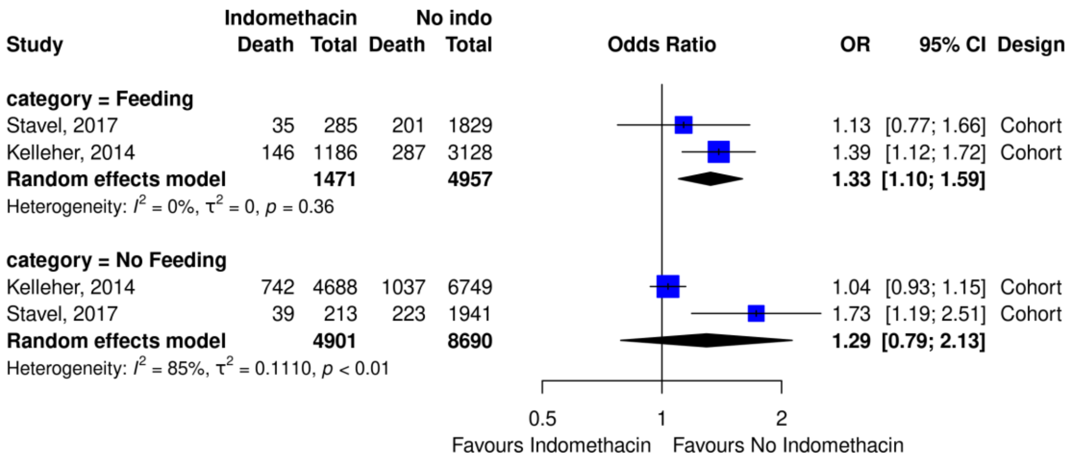

Like other outcomes, premature infants on assisted feeding and taking Indomethacin have higher risk of death before discharge compared to those who didn't take Indomethacin.

For those on no feeding, there is borderline evidence that there is an increased risk for those taking Indomethacin vs not, however the heterogeneity is high so don't report the pooled estimate.

The prevalence of death before discharge in premature infants taking Ibuprofen, stratified by the drug's route. (Indirect comparison)

We only have one study in the Oral group so we cannot compare between the two groups' proportions. The figure below for illustration only.

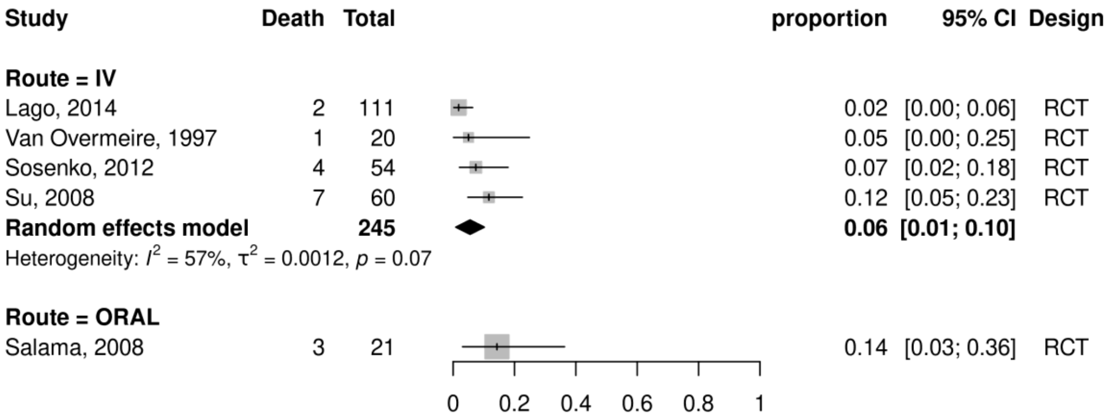

Antenatal Medications (Steroids, indomethacin and Mg sulphate) Analyses

Antenatal steroids

15 studies reported the use of antenatal steroids.
Of 3.0916^{4} patients who took Indomethacin, 2.052^{4} took antenatal steroids (66%).

Antenatal Indomethacin

6 studies reported the use of antenatal Indomethacin with the use of postnatal Indomethacin. Of 672 children who took postnatal Indomethacin, 186 also, took antenatal Indomethacin (28 %).

Mg sulphate

3 studies reported the use of antenatal Mg sulphate in children who took postnatal Indomethacin. Of 109 patients who took postnatal Indomethacin, 52 took antenatal Mg sulphate (48%).

Antenatal steroids and SIP (cases vs controls)

Figure 29: Forest plot: Antenatal steroids between SIP and controls

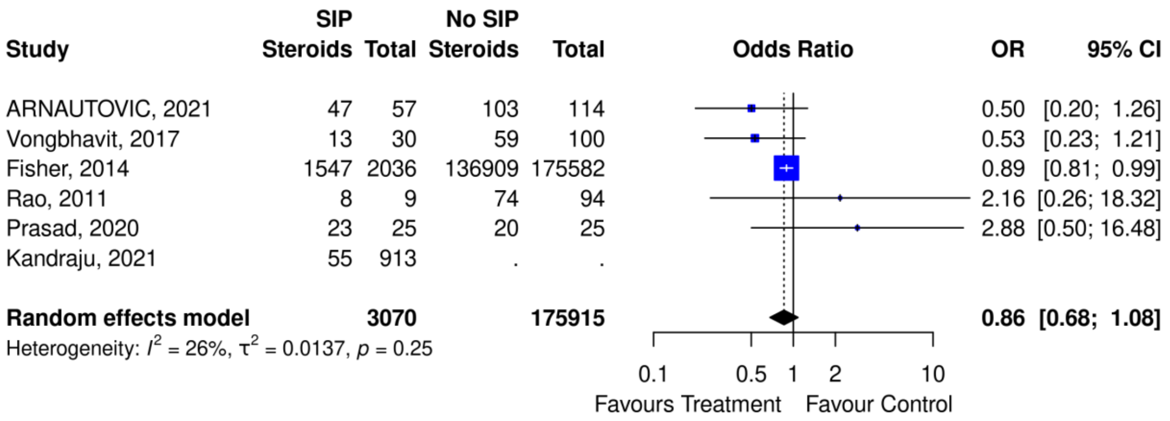

There is no evidence that the odds of antenatal steroids is higher in cases of SIP compared to controls. To note the larger study by Fisher, 2014 which had the largest sample size and the narrowest CI, showed that SIP cases had lower odds of antenatal steroid by 11% (0.89, 95% CI: 0.81, 0.99).

Post-natal steroids

Figure 30: Showing the pooled prevalence of SIP in patients taking Indomethacin with postnatal steroids.

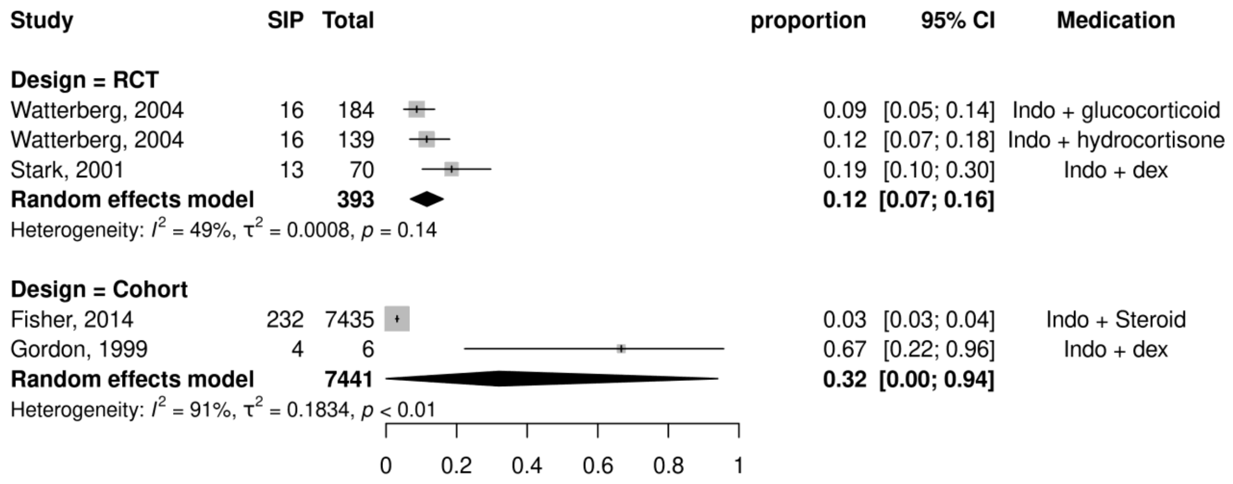

dex= Dexamethasone. Don't provide the pooled estimate for the cohort studies because of the high heterogeneity.

Analysis related to Indirect Feeding groups in studies of Indomethacin and Ibuprofen

Figure 31: Pooled Prevalence of SIP for infants on feeding vs no feeding who are taking Indomethacin (studies are not comparative).

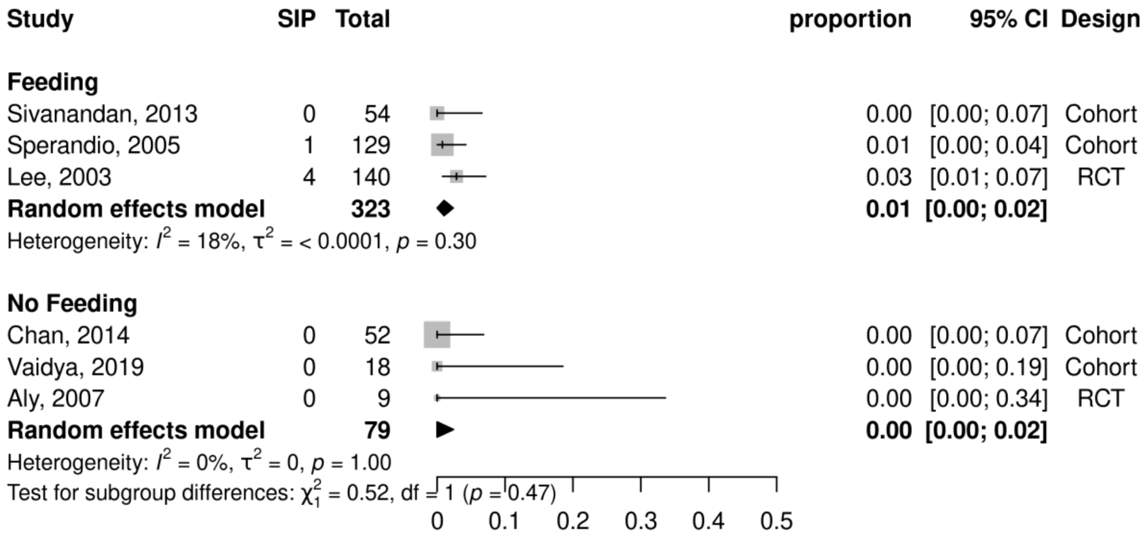

The test for subgroup difference shows there is no difference between the 2 groups.

Pooled Prevalence of NEC for infants on feeding vs no feeding who are taking Indomethacin (studies are not comparative).

We have only one study in the no feeding group, so we didn't test for difference and the figure for illustration only.

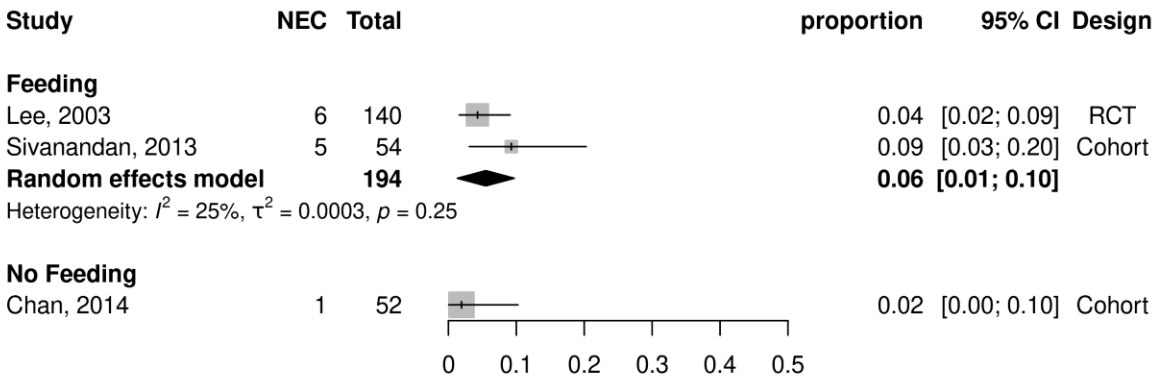

Oliguria and indirect feeding

We have only 2 studies both in the feeding group.

Death before discharge and indirect feeding
We have only 2 studies; one in the feeding and the other in the no feeding so we cannot compare.

For IBU with indirect feeding comparison

We have only one study in the no feeding group and 8 studies in the feeding group. So we cannot pool the prevalence for the no feeding group and compare.

Table 1: Descriptive of the population that are taking different NSAIDS.

I didn't stratify by indo vs no Indo or any other NSAIDS?

Variable	Medication; N	Medication; n (%)	No Medication; N	No medication; n (%)
**Retinopathy of prematurity**	12255	1916 (15.63)	20581	1493 (7.25)
**Oxygen at 36 weeks**	13638	5900 (43.26)	22833	6346 (27.79)
**Intraventricular Hemorrhage**	17099	2326 (13.6)	24191	1991 (8.23)

```
## `summarise()` has grouped output by 'Year'. You can override using the `.groups` argumen
t.
```

Figure 32: Incidence proportion of SIP in the included studies across the years for both INDO and IBU, with a smoothing line (weighted by sample size).

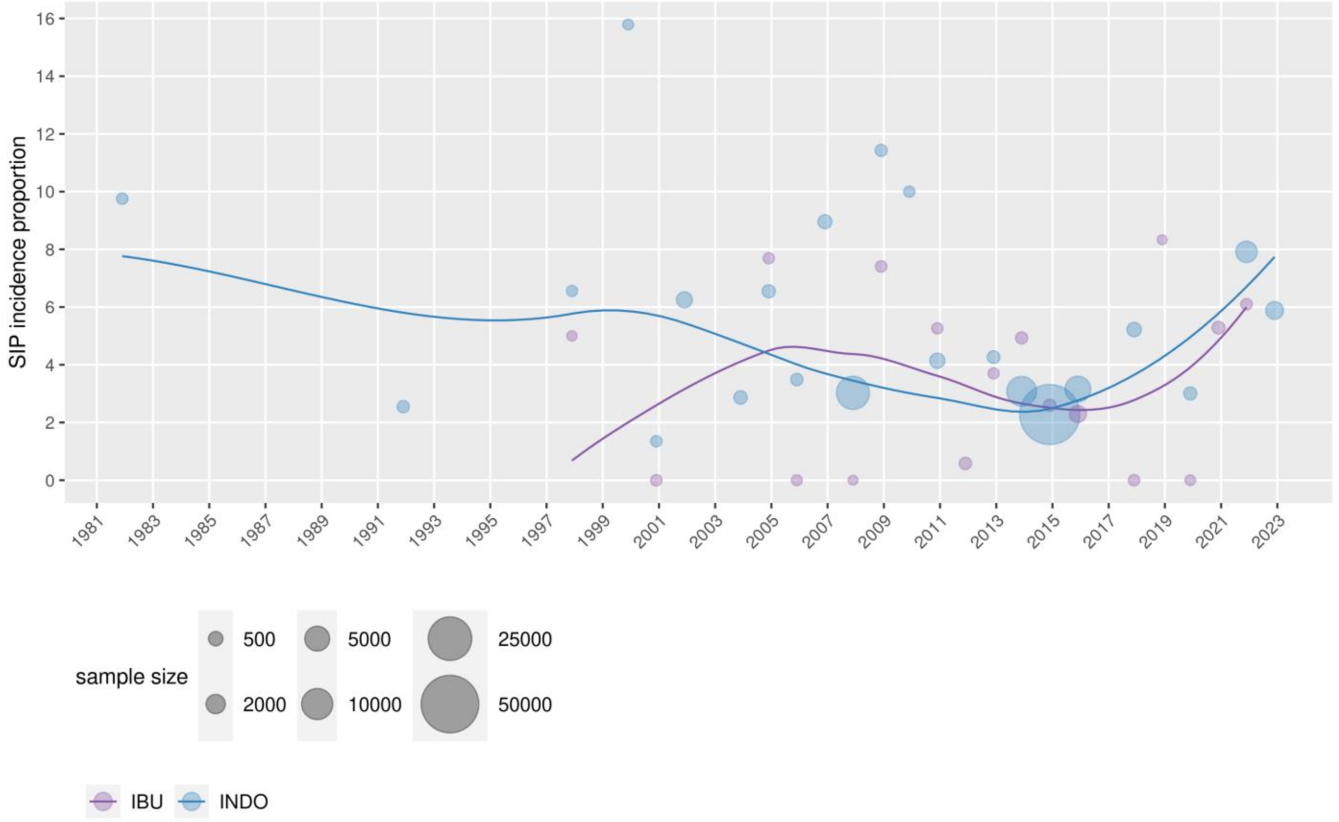

Comparing the proportion of SIP in patients taking Ibuprofen across RCT studies from 2012 and after vs before.

Subgroup meta-analysis of SIP for patients taking IBU comparing the pooled estimate between (2012 and after studies vs before). RCT studies only.

For the studies by Hochwald, we used only the IBU alone arm.

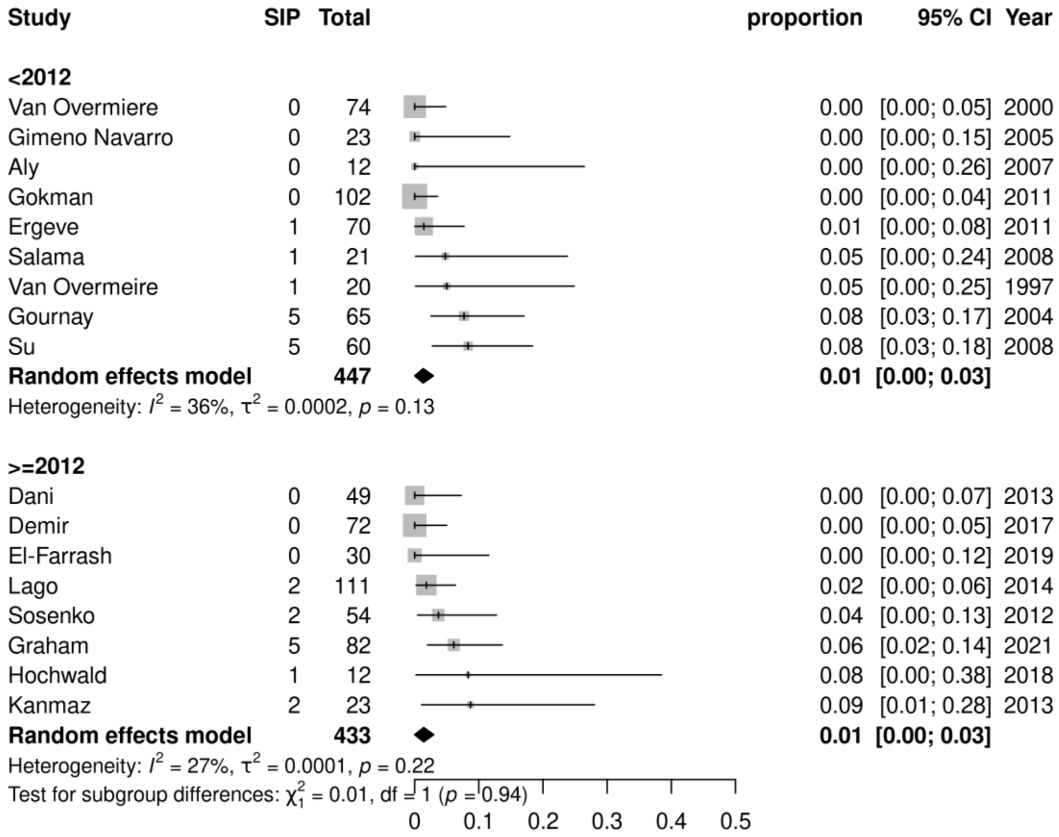

The test for subgroup difference in RCT studies shows that there is no difference in the pooled estimate of SIP in patients taking Ibuprofen between studies <2012 and after (p value = 0.94).

We also conducted the same comparison using Fisher's exact test and the p value was = 0.92.
