# Supplementary material for: Use of NSAIDs and acetaminophen and risk of spontaneous intestinal perforations in premature infants: a systematic review and meta-analysis
Source: Front Pediatr. 2024 Nov 22;12:1450121. doi: 10.3389/fped.2024.1450121 (PMC11620902; doi:10.3389/fped.2024.1450121)
Supplement: Supplementary file 1 [file Presentation1.zip › Supplementary Data Sheet 2.pdf]

Supplementary Material - Data extraction points

General paper demographics:

- Manuscript code (each study was assigned a number)
- Author
- Year of publication
- Study design (RCT, cohort, case control)
- Comparison/ question proposed in the paper

Postnatal medication of interest:

- Which medication was used? Indomethacin, ibuprofen, acetaminophen
- Dose
- Rate
- Route
- Frequency
- Total number of doses
- Number of courses

For antenatal medications including indomethacin, steroids or magnesium sulfate the following was extracted when available:

- Control group
    - Antenatal medication of interest in the control group and control group total
    - Antenatal medication of interest in the control group + SIP and Antenatal medication in the control group total
- Intervention group
    - Antenatal medication of interest in the intervention group and intervention group total
    - Antenatal medication + postnatal medication of interest and total postnatal medication of interest
    - Antenatal medication + postnatal medication of interest + had SIP and Antenatal medication + postnatal medication of interest

For postnatal steroids the following information was extracted when available:

- Control group
    - Postnatal steroid of interest in the control group and control group total
    - Postnatal steroid of interest in the control group + SIP and Postnatal steroid in the control group total
- Intervention group

- Postnatal steroids in the intervention group and intervention group total
- Postnatal steroids + postnatal medication of interest and total postnatal medication of interest
- Postnatal steroids + postnatal medication of interest + had SIP and Postnatal steroids + postnatal medication of interest

Feed regime information was extracted when available:

- Did patients receive feeds yes or no
- If feed any increases in feeds
- If the study design explored relationship of SIP and feeds extraction done fully for all regime options studied

For each of the following outcomes the definition for each outcome and the presence for both control and intervention groups was extracted:

- SIP
- NEC – Bell stage 2 or greater
- AKI as denoted by oliguria
- Death before discharge
- ROP – Any stage
- IVH – Any grade
- BPD - At 36 weeks postmenstrual age
