# Supplementary material for: Use of NSAIDs and acetaminophen and risk of spontaneous intestinal perforations in premature infants: a systematic review and meta-analysis
Source: Front Pediatr. 2024 Nov 22;12:1450121. doi: 10.3389/fped.2024.1450121 (PMC11620902; doi:10.3389/fped.2024.1450121)
Supplement: Supplementary file 1 [file Presentation1.zip › Supplementary Data Sheet 1.pdf]

Supplemental Material – Search strategy

The following is the list of dates that searches were conducted and the rationale.

- November 2016 – Original search*
- Feb 29 2020 - Update
- February 19 2021 – Expanded search terms*
- May 2022 - Update
- September 30th, 2022 - Update
- September 9th, 2023 - Systematic review search and snowballing

* Below is a list of the search strategies employed

<u>November 2016 – Original search strategies</u>

Medline
1 infant, newborn/ or exp infant, low birth weight/ or exp infant, premature/ (549621)
2 Infant, Premature, Diseases/ (19289)
3 ((premature or preterm) adj2 (bab* or infant* or neonat*)).tw. (43537)
4 or/1-3 (555955)
5 Ibuprofen/ (7521)
6 ibuprofen.tw. (10817)
7 brufen.tw. (86)
8 motrin.tw. (75)
9 ibumetin.tw. (5)
10 nuprin.tw. (4)
11 rufen.tw. (1)
12 salprofen.tw. (0)
13 Acetaminophen/ (15721)
14 acetaminophen.tw. (11946)
15 paracetamol.tw. (9442)
16 Acetamidophenol.tw. (110)
17 tylenol.tw. (173)
18 anacin.tw. (4)
19 panadol.tw. (31)
20 datril.tw. (0)
21 acamol.tw. (2)
22 algotropyl.tw. (0)
23 acephen.tw. (7)
24 Indomethacin/ (27635)
25 (indomet* or indocid or indocin or osmosin or metindol or amuno).tw. (35143)
26 or/5-25 (73292)
27 Intestinal Perforation/ (12081)

28 (intestinal adj2 perforat*).tw. (2207)
29 Enterocolitis, Necrotizing/ (2612)
30 (necrot* adj3 enterocolit*).tw. (6197)
31 or/27-30 (19347)
32 4 and 26 and 31 (258)

Embase
33 infant/ or baby/ or newborn/ (1051375)
34 prematurity/ (101946)
35 ((premature or preterm) adj2 (bab* or infant* or neonate*)).ti,ab. (56626)
36 or/33-35 (1083373)
37 ibuprofen/ (42730)
38 ibuprofen.ti,ab. (15286)
39 brufen.ti,ab. (145)
40 motrin.ti,ab. (131)
41 ibumetin.ti,ab. (5)
42 nuprin.ti,ab. (4)
43 rufen.ti,ab. (0)
44 salprofen.ti,ab. (0)
45 paracetamol/ (76152)
46 acetaminophen.ti,ab. (17067)
47 paracetamol.ti,ab. (15424)
48 tylenol.ti,ab. (363)
49 anacin.ti,ab. (5)
50 panadol.ti,ab. (54)
51 datril.ti,ab. (2)
52 acamol.ti,ab. (2)
53 algotropyl.ti,ab. (0)
54 acephen.ti,ab. (6)
55 indometacin/ (75842)
56 (indomet* or indocid or indocin or osmosin or metindol or amuno).ti,ab. (42216)
57 Acetamidophenol.ti,ab. (149)
58 or/37-57 (178171)
59 exp intestine perforation/ (24561)
60 (intestinal adj2 perforat*).ti,ab. (3179)
61 necrotizing enterocolitis/ (8808)
62 (necrot* adj3 enterocolit*).ti,ab. (8205)
63 or/59-62 (35492)
64 36 and 58 and 63 (640)

PubMed
((((((intestinal perforation[MeSH Terms]) OR intestinal perforation[Title/Abstract]) OR enterocolitis, necrotizing[MeSH Terms]) OR necrot* AND enterocolitis[Title/Abstract])) AND

(((((((ibuprofen[MeSH Terms]) OR ibuprofen[Title/Abstract]) OR acetominophen[MeSH Terms]) OR acetominophen[Title/Abstract]) OR indomethacin[MeSH Terms]) OR indomethacin[Title/Abstract]) OR ((indomet*[Title/Abstract] OR indocid[Title/Abstract] OR indocin[Title/Abstract] OR osmosin[Title/Abstract] OR metindol[Title/Abstract] OR amuno))) OR paracetamol[Title/Abstract])) AND (((((infant, newborn[MeSH Terms]) OR infant, low birth weight[MeSH Terms]) OR infant, premature[MeSH Terms]) OR infant, premature, diseases[MeSH Terms]) OR (((premature[Title/Abstract] OR preterm) AND Title/Abstract AND (neonat*[Title/Abstract] OR bab*[Title/Abstract] OR infant*)) AND Title/Abstract))

February 19 2021 – Expanded search strategies

Medline and CENTRAL

1. infant, newborn/ or exp infant, low birth weight/ or exp infant, premature/
2. Infant, Premature, Diseases/
3. ((premature or preterm) adj2 (bab* or infant* or neonat*)).tw.
4. or/1-3
5. Ibuprofen/
6. ibuprofen.tw.
7. brufen.tw.
8. motrin.tw.
9. ibumetin.tw.
10. nuprin.tw.
11. rufen.tw.
12. salprofen.tw.
13. Acetaminophen/
14. acetaminophen.tw.
15. paracetamol.tw.
16. Acetamidophenol.tw.
17. tylenol.tw.
18. anacin.tw.
19. panadol.tw.
20. datril.tw.
21. acamol.tw.
22. algotropyl.tw.
23. acephen.tw.
24. Indomethacin/
25. (indomet* or indocid or indocin or osmosin or metindol or amuno).tw.
26. or/5-25
27. Intestinal Perforation/
28. (intestinal adj2 perforat*).tw.
29. Enterocolitis, Necrotizing/
30. (necrot* adj3 enterocolit*).tw.

31. or/27-30
32. 4 and 26 and 31
33. Pneumoperitoneum/
34. ((multifocal or multi-focal or intestinal or gastrointestinal or bowel or spontaneous or isolated or localized or focal) adj2 perforat*).tw.
35. Pneumoperitoneum.tw.
36. 33 or 34 or 35
37. 4 and 26 and 36

Embase
1. infant/ or baby/ or newborn/
2. prematurity/
3. ((premature or preterm) adj2 (bab* or infant* or neonate*)).ti,ab.
4. or/1-3
5. ibuprofen/
6. ibuprofen.ti,ab.
7. brufen.ti,ab.
8. motrin.ti,ab.
9. ibumetin.ti,ab.
10. nuprin.ti,ab.
11. rufen.ti,ab.
12. salprofen.ti,ab.
13. paracetamol/
14. acetaminophen.ti,ab.
15. paracetamol.ti,ab.
16. tylenol.ti,ab.
17. anacin.ti,ab.
18. panadol.ti,ab.
19. datril.ti,ab.
20. acamol.ti,ab.
21. algotropyl.ti,ab.
22. acephen.ti,ab.
23. indometacin/
24. (indomet* or indocid or indocin or osmosin or metindol or amuno).ti,ab.
25. Acetamidophenol.ti,ab.
26. or/5-25
27. exp intestine perforation/
28. (intestinal adj2 perforat*).ti,ab.
29. necrotizing enterocolitis/
30. (necrot* adj3 enterocolit*).ti,ab.
31. or/27-30
32. 4 and 26 and 31
33. ((multifocal or multi-focal or intestinal or gastrointestinal or bowel or spontaneous or isolated or localized or focal) adj2 perforat*).tw.

34. pneumoperitoneum/
35. pneumoperitoneum.tw.
36. 33 or 34 or 35
37. 4 and 26 and 36

LILACS
perforation AND (neonate or neonates or baby or babies or infant or infants or preterm)

ScIELo
perforation AND (neonate or neonates or baby or babies or infant or infants or preterm)

<u>September 9th, 2023 - Systematic review search and snowballing</u>

1. Systematic review snowballing (23)
   For each article included (including case reports) references were searched. If in the title included either a medication of interest or intestinal perforation a full text screen was performed. Any articles of interest were then subsequently reviewed by two independent reviewers to determine inclusion or exclusion from the study.

2. Systematic review reference check
   Two pubmed searches were performed (on September 9th, 2022) looking for systematic reviews: first using "patent ductus arteriosus" AND (pharm* or Drug*) and secondly using "patent ductus arteriosus" AND (neonate or infant or premature) as search terms. The five most current systematic reviews for each search were selected. The list of included articles for each systematic review were screened. If in the title included either a medication of interest or intestinal perforation a full text screen was performed. Any articles of interest were then subsequently reviewed by two independent reviewers to determine inclusion or exclusion from the study.
