# Supplementary material for: Use of NSAIDs and acetaminophen and risk of spontaneous intestinal perforations in premature infants: a systematic review and meta-analysis
Source: Front Pediatr. 2024 Nov 22;12:1450121. doi: 10.3389/fped.2024.1450121 (PMC11620902; doi:10.3389/fped.2024.1450121)
Supplement: Supplementary file 1 [file Presentation1.zip › Supplementary Table 1 .pdf]

**Supplementary Table 1 – Summary of changed from published protocol to final analysis**

| Section | Published protocol | Final review | Reason for change |
|---|---|---|---|
| Type of study | Inclusion of all case study/ series | Not included in analysis | |
| Type of study | Inclusion of all cohort studies | Did not include studies with no control example all had SIP or all cases exposed to drug or paper only compared SIP to NEC | |
| Secondary outcomes | Inclusion of PDA as an outcome | Not recorded | Many papers explore PDA treatment creating selection bias |
| Secondary outcomes | NEC: Radiological (pneumatosis intestinalis, distended intestinal loops, thickened walls, portal venous gas, pneumoperitoneum) or surgical evidence of NEC with or without the Bell staging | NEC: Radiological (pneumatosis intestinalis, distended intestinal loops, thickened walls, portal venous gas, pneumoperitoneum) or surgical evidence of NEC or without the Bell stage 2 or greater | Pathology more specific to prevent any possible overlap with SIP |
| Secondary outcomes | Acute kidney injury | Oliguria | Only renal indicator captured was oliguria. Decision was to go with that terminology to prevent erroneously calling acute kidney injury based on one sole marker. |
| Secondary outcomes | Bronchopulmonary dysplasia (BPD): Requirement of oxygen and/or positive pressure at 28 days or 36 weeks postmenstrual age or at time of discharge | Bronchopulmonary dysplasia (BPD): Requirement of oxygen and/or positive pressure at 36 weeks postmenstrual age or at time of discharge if prior to 36 weeks | Consistency that is closer to current definition of BPD |
