# Supplementary material for: Use of NSAIDs and acetaminophen and risk of spontaneous intestinal perforations in premature infants: a systematic review and meta-analysis
Source: Front Pediatr. 2024 Nov 22;12:1450121. doi: 10.3389/fped.2024.1450121 (PMC11620902; doi:10.3389/fped.2024.1450121)
Supplement: Supplementary file 1 [file Presentation1.zip › Supplementary Table 2.pdf]

Supplementary Table 2– Article exclusion list

Study Reference	Reason for exclusion	Search origin
Storring N, Amess P, Aiton N, *et al* PO-0495 Intravenous Ibuprofen (ibu) Vs Continuous Indomethacin-infusion (ind-inf) For Symptomatic Patent Ductus Arteriosus (pda) Treatment In Newborns Archives of Disease in Childhood 2014;99:A410.	Poster abstract	1
Concheiro-Guisan A., Gonzalez Duran M.L., Fernandez-Santamarina I., Pumarada Prieto M., ID840. High-dose ibuprofen for patent ductus closure in vlbw infants: a positive experience of a single centre J Matern Fetal Neonatal Med, 2014; 27(S1): 1–437	Poster abstract	1
Fischer A., Durand M., Vachon L., Barton L., Cayabyab, R. Differences in clinical presentation of preterm infants (birth weight <= 1250 grams) with spontaneous intestinal perforation. Journal of Investigative Medicine & Volume 61, Number 1, January 2013	Poster abstract	1
Garg A, Bhatia V, Dasireddy V. A059 Retrospective cohort study comparing safety and efficacy of intravenous ibuprofen and indomethacin for patent ductus arteriosus (pda) closure. Journal of Paediatrics and Child Health 51 (Suppl. 1) (2015), 1–138.	Poster abstract	1
M Munoz-Garcia M, Munoz-Garcia E, Munoz Garcia AJ, Alados-Arboledas FJ, Exposito-Montes JF, De La Cruz-Moreno J. P1583. Clinical characteristics of preterm infants with low birth weight and patent ductus arteriosus treated with ibuprofen. European Journal of Heart Failure © 2015 European Society of Cardiology, 17 (Suppl. 1), 5–441.	Poster abstract	1
O'Hare SS, Tooley JR. 58. Spontaneous intestinal perforation in preterm infants: experience of a uk surgical centre. Intensive Care Med (2011) 37 (Suppl 2):S315–S442.	Poster abstract	1
Parra Llorca, A., Zanon Ortiz, S., Aguado Lozano, V., Aguar Carrascosa, M., Gimeno Navarro, A., Izquierdo Macian, I. O-0129. Spontaneous intestinal perforation caused by ibuprofen during the treatment of patent ductus arteriosus. J. Perinat. Med. 43 (2015)	Poster abstract	1
Pharm J. 117. Early Use of Indomethacin for Prevention of Intraventricular Hemorrhage: a case study highlighting the importance of secondary outcomes. PHARMACOTHERAPY Volume 35, Number 11, 2015	Poster abstract	1
Briscoe A, Dimery R, Wach R. G145(P). indomethacin vs ibuprofen to treat patent ductus arteriosus (pda) in neonates <31 weeks gestation reduced the incidence of necrotizing enterocolitis (nec). Arch Dis Child 2015;100(Suppl 3):A1–A288.	Poster abstract	1
Koshinaga T, Gotoh H, Sugito K, Ikeda T, Hagiwara N, Tomita R. Spontaneous localized intestinal perforation and intestinal dilatation in very-low-birthweight infants. Acta Paediatr. 2006 Nov;95(11):1381-8. doi: 10.1080/08035250600617123. PMID: 17062464	No control group	1
Kühl G, Wille L, Bolkenius M, Seyberth HW. Intestinal perforation associated with indomethacin treatment in	Case report/ series	1

premature infants. Eur J Pediatr. 1985 Jan;143(3):213-6. doi: 10.1007/BF00442142. PMID: 3987718.		
Holland AJ, Shun A, Martin HC, Cooke-Yarborough C, Holland J. Small bowel perforation in the premature neonate: congenital or acquired? Pediatr Surg Int. 2003 Aug;19(6):489-94. doi: 10.1007/s00383-003-0967-8. Epub 2003 May 13. PMID: 12748799.	No control group	1
Alpan G, Eyal F, Vinograd I, Udassin R, Amir G, Mogle P, Glick B. Localized intestinal perforations after enteral administration of indomethacin in premature infants. J Pediatr. 1985 Feb;106(2):277-81. doi: 10.1016/s0022-3476(85)80305-x. PMID: 3968618.	Case report/ series	1
Scholz TD, McGuinness GA. Localized intestinal perforation following intravenous indomethacin for patent ductus arteriosus. J Pediatr Gastroenterol Nutr. 1988 Sep-Oct;7(5):773-5. doi: 10.1097/00005176-198809000-00026. PMID: 3183883.	Case report/ series	1
Tatli MM, Kumral A, Duman N, Demir K, Gurcu O, Ozkan H. Spontaneous intestinal perforation after oral ibuprofen treatment of patent ductus arteriosus in two very-low-birthweight infants. Acta Paediatr. 2004 Jul;93(7):999-1001. doi: 10.1111/j.1651-2227.2004.tb02702.x. PMID: 15303820.	Case report/ series	1
Attridge JT, Clark R, Walker MW, Gordon PV. New insights into spontaneous intestinal perforation using a national data set: (2) two populations of patients with perforations. J Perinatol. 2006 Mar;26(3):185-8. doi: 10.1038/sj.jp.7211439. PMID: 16493433.	Other (Study from same data set at antoher included article)	1
Chang HY, Lung HL, Li ST, Lin CY, Lee HC, Lee CH, Hung HF, Peng CC. Outcomes of Prophylactic Indomethacin for Extremely Low Birth Weight Infants. HK J Paediatr (new series) 2015;20:62-70.	SIP not a separate outcome	1
Clyman R, Wickremasinghe A, Jhaveri N, Hassinger DC, Attridge JT, Sanocka U, Polin R, Gillam-Krakauer M, Reese J, Mammel M, Couser R, Mulrooney N, Yanowitz TD, Derrick M, Jegatheesan P, Walsh M, Fujii A, Porta N, Carey WA, Swanson JR; Ductus Arteriosus Feed or Fast with Indomethacin or Ibuprofen (DAFFII) Investigators. Enteral feeding during indomethacin and ibuprofen treatment of a patent ductus arteriosus. J Pediatr. 2013 Aug;163(2):406-11. doi: 10.1016/j.jpeds.2013.01.057. Epub 2013 Mar 6. PMID: 23472765; PMCID: PMC3683087.	SIP not a separate outcome	1
Dornelles LV, Corso AL, Silveira Rde C, Procianoy RS. Comparison of two dose regimens of ibuprofen for the closure of patent ductus arteriosus in preterm newborns. J Pediatr (Rio J). 2016 May-Jun;92(3):314-8. doi: 10.1016/j.jped.2015.09.009. Epub 2016 Mar 3. PMID: 26946966.	SIP not a separate outcome	1
Grosfeld JL, Chaet M, Molinari F, Engle W, Engum SA, West KW, Rescorla FJ, Scherer LR 3rd. Increased risk of necrotizing enterocolitis in premature infants with patent ductus arteriosus treated with indomethacin. Ann Surg. 1996 Sep;224(3):350-5; discussion 355-7. doi: 10.1097/00000658-199609000-00011. PMID: 8813263; PMCID: PMC1235380.	SIP not a separate outcome	1
Lee CH, Chen HN, Tsao LY, Hsiao CC, Lee ML. Oral ibuprofen versus intravenous indomethacin for closure of patent ductus arteriosus in very low birth weight infants. Pediatr Neonatol.	SIP not a separate outcome	1

2012 Dec;53(6):346-53. doi: 10.1016/j.pedneo.2012.08.011. Epub 2012 Sep 29. PMID: 23276438.		
Lin XZ, Chen HQ, Zheng Z, Li YD, Lai JD, Huang LH. [Therapeutic effect of early administration of oral ibuprofen in very low birth weight infants with patent ductus arteriosus]. Zhongguo Dang Dai Er Ke Za Zhi. 2012 Jul;14(7):502-5. Chinese. PMID: 22809601.	SIP not a separate outcome	1
Linder N, Bello R, Hernandez A, Rosen C, Birk E, Sirota L, Pushkov Y, Klinger G. Treatment of patent ductus arteriosus: indomethacin or ibuprofen? Am J Perinatol. 2010 May;27(5):399-404. doi: 10.1055/s-0029-1243315. Epub 2009 Dec 10. PMID: 20013578.	SIP not a separate outcome	1
Mekkhayai Y, Sornsuvit C, Preedisripipat K, Pongpittayut S. Effectiveness and safety of high dose oral ibuprofen versus standard dose for treatment of preterm infants with patent ductus arteriosus. Int J Pharm Pharm Sci, Vol 7, Issue 10, 338-341.	SIP not a separate outcome	1
Pourarian S, Takmil F, Cheriki S, Amoozgar H. The Effect of Oral High-dose Ibuprofen on Patent Ductus Arteriosus Closure in Preterm Infants. Am J Perinatol. 2015 Oct;32(12):1158-63. doi: 10.1055/s-0035-1551671. Epub 2015 May 22. PMID: 26007314.	SIP not a separate outcome	1
Terrin G, Conte F, Scipione A, Bacchio E, Conti MG, Ferro R, Ventriglia F, De Curtis M. Efficacy of paracetamol for the treatment of patent ductus arteriosus in preterm neonates. Ital J Pediatr. 2014 Feb 20;40(1):21. doi: 10.1186/1824-7288-40-21. PMID: 24555510; PMCID: PMC3940001.	SIP not a separate outcome	1
Koehne PS, Bein G, Alexi-Meskhishvili V, Weng Y, Bührer C, Obladen M. Patent ductus arteriosus in very low birthweight infants: complications of pharmacological and surgical treatment. J Perinat Med. 2001;29(4):327-34. doi: 10.1515/JPM.2001.047. PMID: 11565202.	SIP not a separate outcome	1
Little DC, Pratt TC, Blalock SE, Krauss DR, Cooney DR, Custer MD. Patent ductus arteriosus in micropreemies and full-term infants: the relative merits of surgical ligation versus indomethacin treatment. J Pediatr Surg. 2003 Mar;38(3):492-6. doi: 10.1053/jpsu.2003.50086. PMID: 12632374.	Other (Unclear Methodology)	1
Madan J, Fiascone J, Balasubramanian V, Griffith J, Hagadorn JI. Predictors of ductal closure and intestinal complications in very low birth weight infants treated with indomethacin. Neonatology. 2008;94(1):45-51. doi: 10.1159/000113058. Epub 2008 Jan 15. PMID: 18196930.	No control group	1
Buchheit JQ, Stewart DL. Clinical comparison of localized intestinal perforation and necrotizing enterocolitis in neonates. Pediatrics. 1994 Jan;93(1):32-6. PMID: 8265320.	No control group (SIP versus NEC)	1
Hwang H, Murphy JJ, Gow KW, Magee JF, Bekhit E, Jamieson D. Are localized intestinal perforations distinct from necrotizing enterocolitis? J Pediatr Surg. 2003 May;38(5):763-7. doi: 10.1016/jpsu.2003.50162. PMID: 12720189.	No control group (SIP versus NEC)	1
Pumberger W, Mayr M, Kohlhauser C, Weninger M. Spontaneous localized intestinal perforation in very-low-birth-weight infants: a distinct clinical entity different from	No control group (SIP versus NEC)	1

necrotizing enterocolitis. J Am Coll Surg. 2002 Dec;195(6):796-803. doi: 10.1016/s1072-7515(02)01344-3. PMID: 12495312.		
Tarrado X, Castañón M, Thió M, Valderas JM, Garcia Aparicio L, Morales L. Comparative study between isolated intestinal perforation and necrotizing enterocolitis. Eur J Pediatr Surg. 2005 Apr;15(2):88-94. doi: 10.1055/s-2004-821255. PMID: 15877256.	No control group (SIP versus NEC)	1
Gordon PV, Clark R, Swanson JR, Spitzer A. Can a national dataset generate a nomogram for necrotizing enterocolitis onset? J Perinatol. 2014 Oct;34(10):732-5. doi: 10.1038/jp.2014.137. Epub 2014 Jul 31. PMID: 25078862.	No control group (SIP versus NEC)	1
Tefft RG. The impact of an early Ibuprofen treatment protocol on the incidence of surgical ligation of the ductus arteriosus. Am J Perinatol. 2010 Jan;27(1):83-90. doi: 10.1055/s-0029-1239492. Epub 2009 Sep 26. PMID: 19784912.	No SIP	1
Yadav S, Agarwal S, Maria A, Dudeja A, Dubey NK, Anand P, Yadav DK. Comparison of oral ibuprofen with oral indomethacin for PDA closure in Indian preterm neonates: a randomized controlled trial. Pediatr Cardiol. 2014 Jun;35(5):824-30. doi: 10.1007/s00246-014-0861-2. Epub 2014 Jan 17. PMID: 24435507.	No SIP	1
Jakaitis BM, Bhatia AM. Definitive peritoneal drainage in the extremely low birth weight infant with spontaneous intestinal perforation: predictors and hospital outcomes. J Perinatol. 2015 Aug;35(8):607-11. doi: 10.1038/jp.2015.23. Epub 2015 Apr 9. PMID: 25856761.	No control group	1
Paquette L, Friedlich P, Ramanathan R, Seri I. Concurrent use of indomethacin and dexamethasone increases the risk of spontaneous intestinal perforation in very low birth weight neonates. J Perinatol. 2006 Aug;26(8):486-92. doi: 10.1038/sj.jp.7211548. Epub 2006 Jun 22. PMID: 16791261.	Other (data too entangles, cannot extract SIP)	1
Yoshimoto S, Sakai H, Ueda M, Yoshikata M, Mizobuchi M, Nakao H. Prophylactic indomethacin in extremely premature infants between 23 and 24 weeks gestation. Pediatr Int. 2010 Jun;52(3):374-7. doi: 10.1111/j.1442-200X.2009.02977.x. Epub 2009 Oct 14. PMID: 19843236.	SIP not a separate outcome	1
Pietz J, Achanti B, Lilien L, Stepka EC, Mehta SK. Prevention of necrotizing enterocolitis in preterm infants: a 20-year experience. Pediatrics. 2007 Jan;119(1):e164-70. doi: 10.1542/peds.2006-0521. Epub 2006 Dec 4. PMID: 17145901.	No SIP	1
Tsao PC, Chen SJ, Yang CF, Lee YS, Jeng MJ, Soong WJ, Lee PC, Lu JH, Hwang B, Tang RB. Comparison of intravenous and enteral indomethacin administration for closure of patent ductus arteriosus in extremely-low-birth-weight infants. J Chin Med Assoc. 2010 Jan;73(1):15-20. doi: 10.1016/s1726-4901(10)70016-2. PMID: 20103486.	SIP not a separate outcome	1
Tammela O, Ojala R, Iivainen T, Lautamatti V, Pokela ML, Janas M, Koivisto M, Ikonen S. Short versus prolonged indomethacin therapy for patent ductus arteriosus in preterm infants. J Pediatr. 1999 May;134(5):552-7. doi: 10.1016/s0022-3476(99)70239-8. PMID: 10228288.	SIP not a separate outcome	1

Rao R, Bryowsky K, Mao J, Bunton D, McPherson C, Mathur A. Gastrointestinal complications associated with ibuprofen therapy for patent ductus arteriosus. J Perinatol. 2011 Jul;31(7):465-70. doi: 10.1038/jp.2010.199. Epub 2011 Jan 20. PMID: 21252965.	No control group	1
Louis D, Torgalkar R, Shah J, Shah PS, Jain A. Enteral feeding during indomethacin treatment for patent ductus arteriosus: association with gastrointestinal outcomes. J Perinatol. 2016 Jul;36(7):544-8. doi: 10.1038/jp.2016.11. Epub 2016 Feb 25. PMID: 26914015.	SIP not separate outcome	2
Liebowitz M, Clyman RI. Prophylactic Indomethacin Compared with Delayed Conservative Management of the Patent Ductus Arteriosus in Extremely Preterm Infants: Effects on Neonatal Outcomes. J Pediatr. 2017 Aug;187:119-126.e1. doi: 10.1016/j.jpeds.2017.03.021. Epub 2017 Apr 7. PMID: 28396025; PMCID: PMC5533630.	SIP not separate outcome	2
Sadeghi-Moghaddam P, Arjmandnia MH, Heidari A, Mohagheghi-Kamal SM, Aghaali M. Comparison of Therapeutic Effects and Side Effects of Oral Ibuprofen and Indomethacin on the Closure of Patent Ductus Arteriosus in Premature Infants. J Babol Univ Med Sci; 19(9); Sep 2017; PP: 7-12	SIP not separate outcome	2
Erdeve O, Okulu E, Atasay B, Arsan S, On Behalf Of Interpda Study Group. Early versus conservative treatment: Preliminary data of the national registry on treatment option and its timing in patent ductus arteriosus. Abstracts of the 26th European Workshop on Neonatology.	Poster	2
Hundscheid T, Onland W, van Overmeire B, Dijk P, van Kaam AHLC, Dijkman KP, Kooi EMW, Villamor E, Kroon AA, Visser R, Vijlbrief DC, de Tollenaer SM, Cools F, van Laere D, Johansson AB, Hocq C, Zecic A, Adang E, Donders R, de Vries W, van Heijst AFJ, de Boode WP. Early treatment versus expectative management of patent ductus arteriosus in preterm infants: a multicentre, randomised, non-inferiority trial in Europe (BeNeDuctus trial). BMC Pediatr. 2018 Aug 4;18(1):262. doi: 10.1186/s12887-018-1215-7. PMID: 30077184; PMCID: PMC6090763.	Other No data (Study Protocol)	2
Hwee G, Wu A, Bracamonte A, Chun B, Chadwick J, Lee K, Shayegh O, Goel T, Afghani B. 123. Does indomethacin used for treatment of patent ductus arteriousus (pda) lead to necrotizing enterocolitis and/or intestinal perforation in premature infants? J Investig Med 2018;66:62–287	Poster for systematic review	2
Jung P, Rickards ED, Deming D. 64. patent ductus arteriosus and associated outcomes in extremely preterm infants. J Investig Med 2018;66:62–287	Poster	2
Makoni MM, Milan J , Manfredo A, Chaphekar A. 419. Outcome differences based on management of patent ductus arteriosus in very low birth weight infants in a level iv neonatal intensive care unit. J Investig Med 2018;66:351–640.	Poster	2
Rickards ED, Jung P, Deming D. 62. Outcomes from treatment of the patent ductus arteriosus in very low birth weight neonates. J Investig Med 2018;66:62–287	Poster	2

Clyman RI, Liebowitz M, Kaempf J, Erdeve O, Bulbul A, Håkansson S, Lindqvist J, Farooqi A, Katheria A, Sauberan J, Singh J, Nelson K, Wickremasinghe A, Dong L, Hassinger DC, Aucott SW, Hayashi M, Heuchan AM, Carey WA, Derrick M, Fernandez E, Sankar M, Leone T, Perez J, Serize A; PDA-TOLERATE (PDA: TO LEave it alone or Respond And Treat Early) Trial Investigators. PDA-TOLERATE Trial: An Exploratory Randomized Controlled Trial of Treatment of Moderate-to-Large Patent Ductus Arteriosus at 1 Week of Age. J Pediatr. 2019 Feb;205:41-48.e6. doi: 10.1016/j.jpeds.2018.09.012. Epub 2018 Oct 16. PMID: 30340932; PMCID: PMC6502709.	SIP not separate outcome	2
Dawoud FM, Lawson JG,Shah DS. 419. Unusual case of necrotizing enterocolitis. J Investig Med 2019;67:350–652.	Poster	2
Karabulut B, Paytoncu S. Efficacy and Safety of Oral Paracetamol vs. Oral Ibuprofen in the Treatment of Symptomatic Patent Ductus Arteriosus in Premature Infants. Paediatr Drugs. 2019 Apr;21(2):113-121. doi: 10.1007/s40272-019-00331-z. PMID: 31025304.	No SIP	2
Mainzer G, Hochwald O, Borenstein L, Zucker M, Jubran H, Dinur G, Kugelman A. P003. Adding paracetamol to ibuprofen for treatment of patent ductus arteriosus in preterm infants: A pilot, double blind, randomized, placebo-control trial. Congenital Heart Disease. 2019;14:116–120.	Poster	2
Ohlsson A, Shah SS. Ibuprofen for the prevention of patent ductus arteriosus in preterm and/or low birth weight infants. Cochrane Database Syst Rev. 2020 Jan 27;1(1):CD004213. doi: 10.1002/14651858.CD004213.pub5. PMID: 31985838; PMCID: PMC6984616.	Other No data (Systematic review)	2
Okulu E, Erdeve Ö, Arslan Z., Demirel N., Kaya H., Gökçe İ., Ertuğrul S., Çetinkaya M., Büyükkale G., Atasay B., Özlü F., Şimşek H., Çelik Y., Özkan H., Köksal N. , Akcan B. 10, Türkmen M. 10, Çelik K. 11, Armangil D., Bülbül A., Tekgündüz K., Öncel M., Tüzün F., Ergenekon E., Ergin H., Arsan S. O1363 - Turkish national registry on treatment option and its timing in patent ductus arteriosus interpda trial. J. Perinat. Med. 2019; 47 (Suppl).	Poster	2
Wong A., Jain A., Jenkins S., Shetthalli M., Nathan M., Goodwin T., Ariti C., Uzun O. P-229 Management of patent ductus arteriosus in preterm babies is not a surgical consideration and can be managed conservatively. Cardiology in the Young: Volume 29 Supplement 1	Poster	2
Effect of intravenous acetaminophen in prevention of patent dactus arteriosus in preterm infants IRCT20111011007763N2 10522735	Clinical trial (not found)	2
The comparison between intravenous acetaminophen versus oral ibuprofen in preterm newborns with patent ductus arteriosus IRCT20190206042639N1	Clinical trial (not found)	2
Patent ductus arteriosus (PDA) treatment in premature infants ISRCTN12302923 closed and paper pulished was Al-Lawama M, Alammori I, Abdelghani T, Badran E. Oral paracetamol versus oral ibuprofen for treatment of patent ductus arteriosus. J Int Med Res. 2018 Feb;46(2):811-818. doi:	Clinical trial (not found)	2

10.1177/0300060517722698. Epub 2017 Sep 14. PMID: 29239259; PMCID: PMC5971505.		
Paracetamol versus Ibuprofen For Closure of Patent Ductus Arteriosus CTRI/2017/10/009989 10514425	Clinical trial (No SIP)	2
Conservative management of ductus in preterm neonates PubMed Identifier CTRI/2018/03/012784 10520990	Clinical trial (not found)	2
AlRyalat SA, Al Oweidat K, Al-Amer A, Khader A, Ajaj A, Alessa Z, Roto A. Perinatal events predicting retinopathy of prematurity in extremely pre-term infants. J Neonatal Perinatal Med. 2020;13(2):261-266. doi: 10.3233/NPM-190336. PMID: 32250325.	Other (Could not extract data)	3
Anttila E, Peltoniemi O, Haumont D, Herting E, ter Horst H, Heinonen K, Kero P, Nykänen P, Oetomo SB, Hallman M. Early neonatal dexamethasone treatment for prevention of bronchopulmonary dysplasia. Randomised trial and meta-analysis evaluating the duration of dexamethasone therapy. Eur J Pediatr. 2005 Aug;164(8):472-81. doi: 10.1007/s00431-005-1645-8. Epub 2005 Apr 28. PMID: 15864643.	SIP not a separate outcome	3
Baud O, Watterberg KL. Prophylactic postnatal corticosteroids: Early hydrocortisone. Semin Fetal Neonatal Med. 2019 Jun;24(3):202-206. doi: 10.1016/j.siny.2019.04.007. Epub 2019 Apr 20. PMID: 31043325.	Other (No data)	3
Memisoglu A, Alp Ünkar Z, Cetiner N, Akalın F, Ozdemir H, Bilgen HS, Ozek E. Ductal closure with intravenous paracetamol: a new approach to patent ductus arteriosus treatment. J Matern Fetal Neonatal Med. 2016 Mar;29(6):987-90. doi: 10.3109/14767058.2015.1029912. Epub 2015 Apr 2. PMID: 25781500.	No SIP	3
Seri I. Management of hypotension and low systemic blood flow in the very low birth weight neonate during the first postnatal week. J Perinatol. 2006 May;26 Suppl 1:S8-13; discussion S22-3. doi: 10.1038/sj.jp.7211464. PMID: 16625228.	Other (No data)	3
Soll, R.F., Vermont Oxford Network Steroid Study Group. Early postnatal dexamethasone therapy for the prevention of chronic lung disease. Pediatrics. 2001 Sep;108(3):741-8. doi: 10.1542/peds.108.3.741. PMID: 11533345.	Other (Cant tell which SIP had meds)	3
Shaffer ML, Baud O, Lacaze-Masmonteil T, Peltoniemi OM, Bonsante F, Watterberg KL. Effect of Prophylaxis for Early Adrenal Insufficiency Using Low-Dose Hydrocortisone in Very Preterm Infants: An Individual Patient Data Meta-Analysis. J Pediatr. 2019 Apr;207:136-142.e5. doi: 10.1016/j.jpeds.2018.10.004. Epub 2018 Nov 8. PMID: 30416014.	Other (Could not separate out SIP + MED)	3
Paracetamol versus Ibuprofen For Closure of Patent Ductus Arteriosus http://www.who.int/trialsearch/Trial2.aspx?TrialID=CTRI 2017;10(009989):2017	Clinical trial	3
Comparison of feeding or not feeding the premature babies when they are given oral drug ibuprofen and evaluate the time to reach full feeds in two groups http://www.who.int/trialsearch/Trial2.aspx?TrialID=CTRI 2020;03(023945): 2020	Clinical trial	3

Prophylactic treatment of the ductus arteriosus in preterm infants by paracetamol http://www.who.int/trialsearch/Trial2.aspx?TrialID=EUCTR2019 2019;():2019	Clinical trial	3
Paracetamol versus ibuprofen in preterm infants with a hemodynamically significant patent ductus arteriosus: a randomized clinical trial http://www.who.int/trialsearch/Trial2.aspx?TrialID=EUCTR2015 2015;():2015	Clinical trial (No contact available)	3
Baby-OSCAR (Baby Outcomes after Selective early targeted treatment for Closure of 'open' ductus ARteriosus in preterm babies) http://www.who.int/trialsearch/Trial2.aspx?TrialID=EUCTR2013 2013;():2013	Clinical trial	3
Therapy of patency of ductus arteriosus (PDA) in very low birth weight (VLBW)preterm infants: oral ibuprofen versus intravenous ibuprofen - NEO 12 http://www.who.int/trialsearch/Trial2.aspx?TrialID=EUCTR2010 2010;(): 2010	Clinical trial (Not available)	3
Paracetamol versus ibuprofen, (a drug that represents the standard treatment in this condition) to treat preterm infants with Perstent Ductus Arteriosus unclosure that is without sponatanous clousure at the http://www.who.int/trialsearch/Trial2.aspx?TrialID=EUCTR2013 2013;(): 2013	Clinical trial	3
PREMILOC Trial to Prevent Bronchopulmonary Dysplasia in Very Preterm Neonates https://clinicaltrials.gov/show/NCT00623740 2008;(): 2008	Clinical trial (Can't tell which SIP had Indomethacin)	3
Comparing Ibuprofen And Indomethacin For The Treatment Of The Patent Ductus Arteriosus in Very Premature Babies https://clinicaltrials.gov/show/NCT00470743 2007;(): 2007	Clinical trial (not available)	3
Acetaminophen vs Indomethacin in Treating hsPDA https://clinicaltrials.gov/show/NCT03537144 2018;(): 2018	Clinical trial	3
Marshall TA, Pai S, Reddy PP. Intestinal perforation following enteral administration of indomethacin. J Pediatr. 1985 Sep;107(3):484-5. doi: 10.1016/s0022-3476(85)80541-2. PMID: 3928859.	Case report/ series	3
Foss K. A case report of a low-birth-weight infant with a subcapsular liver hematoma and spontaneous bowel perforation. Adv Neonatal Care. 2004 Apr;4(2):67-78. doi: 10.1016/j.adnc.2004.01.003. PMID: 15138990.	Case report/ series	3
Peitz GJ, Hoie EB, Hoy S, Anderson-Berry A. Repeated bowel perforations with Ibuprofen lysine: a case report. J Pediatr Pharmacol Ther. 2008 Jul;13(3):166-9. doi: 10.5863/1551-6776-13.3.166. PMID: 23055878; PMCID: PMC3462041.	Case report/ series	3
Babayigit A, Ozaydın S, Cetinkaya M, Sander S. Neonatal gastric perforations in very low birth weight infants: a single center experience and review of the literature. Pediatr Surg Int. 2018 Jan;34(1):79-84. doi: 10.1007/s00383-017-4205-1. Epub 2017 Oct 27. PMID: 29079904.	Case report/ series	3
Wolf WM, Snover DC, Leonard AS. Localized intestinal perforation following intravenous indomethacin in premature	Case report/ series	3

infants. J Pediatr Surg. 1989 Apr;24(4):409-10. doi: 10.1016/s0022-3468(89)80284-2. PMID: 2732888.		
Giacoia GP, Azubuike K, Taylor JR. Indomethacin and recurrent ileal perforations in a preterm infant. J Perinatol. 1993 Jul-Aug;13(4):297-9. PMID: 8410386.	Case report/ series	3
Tuteja A, Pournami F, Kolisambeevi AA, Nandakumar A, Prabhakar J, Jain N. Paracetamol for Patent Ductus Arteriosus Closure: High Osmolality of Enteral Form and Spontaneous Intestinal Perforation. Indian J Pediatr. 2020 Sep;87(9):767. doi: 10.1007/s12098-020-03445-2. Epub 2020 Jul 16. PMID: 32671640.	Case report/ series	3
Rayyan M, Myatchin I, Naulaers G, Ali Said Y, Allegaert K, Miserez M. Risk factors for spontaneous localized intestinal perforation in the preterm infant. J Matern Fetal Neonatal Med. 2018 Oct;31(19):2617-2623. doi: 10.1080/14767058.2017.1350161. Epub 2017 Jul 16. PMID: 28671044.	Other	3
Stoodley, N.G., Corbally, M.T. & Speidel, B.D. Simultaneous spontaneous isolated ileal perforation in twins. Pediatr Surg Int 8, 515–516 (1993). https://doi.org/10.1007/BF00180361	Other (No med)	3
Chi, J.G., Kim, K.S. Localized intestinal perforation. The Seoul Journal of Medicine Vol. 35, No.3: 225-229, September 1994	Other (No Med)	3
Trus T, Winthrop AL, Pipe S, Shah J, Langer JC, Lau GY. Optimal management of patent ductus arteriosus in the neonate weighing less than 800 g. J Pediatr Surg. 1993 Sep;28(9):1137-9. doi: 10.1016/0022-3468(93)90148-e. PMID: 8308678.	Other (SIP definition unclear)	3
Simão C, Gonçalves M, Guerreiro O. Peritonite no período neonatal [Peritonitis in the neonatal period]. Acta Med Port. 1998 Dec;11(12):1127-9. Portuguese. PMID: 10192990.	No SIP	3
Barseghyan K, Gayer C, Azhibekov T. Differences in Serum Alkaline Phosphatase Levels in Infants with Spontaneous Intestinal Perforation versus Necrotizing Enterocolitis with Perforation. Neonatology. 2020;117(3):349-357. doi: 10.1159/000509617. Epub 2020 Aug 4. PMID: 32750698.	No control group	3
Brisighelli G, Consonni D, Macchini F, Parente G, Zanini A, Franzini S, Colnaghi M, Mosca F, Leva E. A Single-Center Experience with Very Low Birth Weight Infants and Focal Intestinal Perforation: Comparison of Primary Anastomosis versus Stoma Opening. Eur J Pediatr Surg. 2018 Oct;28(5):426-432. doi: 10.1055/s-0037-1605348. Epub 2017 Aug 24. PMID: 28837996.	No control group	3
Hervás JA, Masip MC, Alomar A, Bregante JI. Localized intestinal perforation after intravenous indomethacin in a premature infant. Helv Paediatr Acta. 1986 Dec;41(5):437-40. PMID: 3818330.	Case report/ series	3
Alvarado-Socarras JL, Gómez-Capacho A, Niño-Tovar MA, Pinilla-Orejana A. Prematuro con perforación intestinal espontánea de presentación muy precoz [Preterm neonate with spontaneous intestinal perforation of very early presentation]. Bol Med Hosp Infant Mex. 2021 Mar 30;78(2):143-147. Spanish. doi: 10.24875/BMHIM.20000054. PMID: 33783460.	Other (No med of interest)	4

Mantle A, Yang MJ, Judkins A, Chanthavong I, Yoder BA, Chan B. Association of Intrapartum Drugs with Spontaneous Intestinal Perforation: A Single-Center Retrospective Review. Am J Perinatol. 2021 Nov 28:10.1055/a-1673-0183. doi: 10.1055/a-1673-0183. Epub ahead of print. PMID: 34666387; PMCID: PMC10435317.	Other (could not tell who has postnatal med and SIP)	4
Turan OM, Driscoll C, Cetinkaya-Demir B, Gabbay-Benziv R, Turan S, Kopelman JN, Harman C. Prolonged early antenatal indomethacin exposure is safe for fetus and neonate. J Matern Fetal Neonatal Med. 2021 Jan;34(2):167-176. doi: 10.1080/14767058.2019.1599351. Epub 2019 Apr 4. PMID: 30905227.	Other (Could not tell which SIP had meds)	4
Travers CP, Gentle S, Freeman AE, Nichols K, Shukla VV, Purvis D, Dolma K, Winter L, Ambalavanan N, Carlo WA, Lal CV. A Quality Improvement Bundle to Improve Outcomes in Extremely Preterm Infants in the First Week. Pediatrics. 2022 Feb 1;149(2):e2020037341. doi: 10.1542/peds.2020-037341. PMID: 35088085; PMCID: PMC9677934.	Other (Could not tell which SIP had meds)	4
Gross M, Poets CF. Lipid enemas for meconium evacuation in preterm infants - a retrospective cohort study. BMC Pediatr. 2021 Oct 18;21(1):454. doi: 10.1186/s12887-021-02905-8. PMID: 34657609; PMCID: PMC8522005.	Other (Could not tell which SIP had meds)	4
Rovers JFJ, Thomissen IJC, Janssen LCE, Lingius S, Wieland BV, Dieleman JP, Niemarkt HJ, van Runnard Heimel PJ. The relationship between antenatal indomethacin as a tocolytic drug and neonatal outcomes: a retrospective cohort study. J Matern Fetal Neonatal Med. 2021 Sep;34(18):2945-2951. doi: 10.1080/14767058.2019.1674807. Epub 2019 Oct 9. PMID: 31597542.	Other (Could not tell which SIP had meds)	4
Silverberg, M. 243 - Reducing Spontaneous Intestinal Perforation Among Extremely Preterm Infants: A Quality Improvement Initiative. PAS 2022.	Poster (Could not tell which SIP had meds)	4
Clyman RI, Jin C, Hills NK. A role for neonatal bacteremia in deaths due to intestinal perforation: spontaneous intestinal perforation compared with perforated necrotizing enterocolitis. J Perinatol. 2020 Nov;40(11):1662-1670. doi: 10.1038/s41372-020-0691-4. Epub 2020 May 20. PMID: 32433511; PMCID: PMC7578088.	No control group (SIP versus NEC)	Review search
Kahn DJ, Gregorisch S, Whitehouse JS, Fisher PD. Delayed diagnosis of spontaneous intestinal perforation among very low birth weight neonates: A single center experience. J Perinatol. 2019 Nov;39(11):1509-1520. doi: 10.1038/s41372-019-0480-0. Epub 2019 Aug 28. PMID: 31462722.	No control group	Review search
Suply E, Leclair MD, Neunlist M, Roze JC, Flamant C. Spontaneous Intestinal Perforation and Necrotizing Enterocolitis: A 16-Year Retrospective Study from a Single Center. Eur J Pediatr Surg. 2015 Dec;25(6):520-5. doi: 10.1055/s-0034-1396418. Epub 2015 Feb 2. PMID: 25643249.	No control group (SIP versus NEC)	Review search
Blakely ML, Lally KP, McDonald S, Brown RL, Barnhart DC, Ricketts RR, Thompson WR, Scherer LR, Klein MD, Letton RW, Chwals WJ, Touloukian RJ, Kurkchubasche AG, Skinner MA, Moss RL, Hilfiker ML; NEC Subcommittee of the NICHD	No control group	Review search

Neonatal Research Network. Postoperative outcomes of extremely low birth-weight infants with necrotizing enterocolitis or isolated intestinal perforation: a prospective cohort study by the NICHD Neonatal Research Network. Ann Surg. 2005 Jun;241(6):984-9; discussion 989-94. doi: 10.1097/01.sla.0000164181.67862.7f. PMID: 15912048; PMCID: PMC1359076.		
Cass DL, Brandt ML, Patel DL, Nuchtern JG, Minifee PK, Wesson DE. Peritoneal drainage as definitive treatment for neonates with isolated intestinal perforation. J Pediatr Surg. 2000 Nov;35(11):1531-6. doi: 10.1053/jpsu.2000.18299. PMID: 11083416.	No control group (SIP versus NEC)	Review search
Rovin JD, Rodgers BM, Burns RC, McGahren ED. The role of peritoneal drainage for intestinal perforation in infants with and without necrotizing enterocolitis. J Pediatr Surg. 1999 Jan;34(1):143-7. doi: 10.1016/s0022-3468(99)90245-2. PMID: 10022160.	No control group (SIP versus NEC)	Review search
Uceda JE, Laos CA, Kolni HW, Klein AM. Intestinal perforations in infants with a very low birth weight: a disease of increasing survival? J Pediatr Surg. 1995 Sep;30(9):1314-6. doi: 10.1016/0022-3468(95)90493-x. PMID: 8523233.	No control group	Review search
Zamir O, Goldberg M, Udassin R, Peleg O, Nissan S, Eyal F. Idiopathic gastrointestinal perforation in the neonate. J Pediatr Surg. 1988 Apr;23(4):335-7. doi: 10.1016/s0022-3468(88)80201-x. PMID: 3385586.	No control group	Review search
Kribs A, Roll C, Göpel W, Wieg C, Groneck P, Laux R, Teig N, Hoehn T, Böhm W, Welzing L, Vochem M, Hoppenz M, Bührer C, Mehler K, Stützer H, Franklin J, Stöhr A, Herting E, Roth B; NINSAPP Trial Investigators. Nonintubated Surfactant Application vs Conventional Therapy in Extremely Preterm Infants: A Randomized Clinical Trial. JAMA Pediatr. 2015 Aug;169(8):723-30. doi: 10.1001/jamapediatrics.2015.0504. PMID: 26053341.	SIP not separate	Review search
Sharma R, Tepas JJ 3rd, Mollitt DL, Pieper P, Wludyka P. Surgical management of bowel perforations and outcome in very low-birth-weight infants (< or =1,200 g). J Pediatr Surg. 2004 Feb;39(2):190-4. doi: 10.1016/j.jpedsurg.2003.10.005. PMID: 14966738.	Other (cannot tell which SIP had med)	Review search
Norton ME, Merrill J, Cooper BA, Kuller JA, Clyman RI. Neonatal complications after the administration of indomethacin for preterm labor. N Engl J Med. 1993 Nov 25;329(22):1602-7. doi: 10.1056/NEJM199311253292202. PMID: 8232428.	SIP not separate	Review search
Ojala R, Ikonen S, Tammela O. Perinatal indomethacin treatment and neonatal complications in preterm infants. Eur J Pediatr. 2000 Mar;159(3):153-5. doi: 10.1007/s004310050040. PMID: 10664225.	SIP not separate	Review search
Harris JP, Merritt TA, Alexson CG, Longfield L, Manning JA. Parenteral indomethacin for closure of the patent ductus arteriosus. Clinical experience with 67 preterm infants. Am J Dis Child. 1982 Nov;136(11):1005-8. doi: 10.1001/archpedi.1982.03970470049014. PMID: 7124692.	No SIP	Review search

Tatekawa Y, Muraji T, Imai Y, Nishijima E, Tsugawa C. The mechanism of focal intestinal perforations in neonates with low birth weight. Pediatr Surg Int. 1999;15(8):549-52. doi: 10.1007/s003830050668. PMID: 10631731.	Case report/ series	Review search
Huang SF, Vacanti J, Kozakewich H. Segmental defect of the intestinal musculature of a newborn: evidence of acquired pathogenesis. J Pediatr Surg. 1996 May;31(5):721-5. doi: 10.1016/s0022-3468(96)90687-9. PMID: 8861493.	Case report/ series	Review search
Izraeli S, Freud E, Mor C, Litwin A, Zer M, Merlob P. Neonatal intestinal perforation due to congenital defects in the intestinal muscularis. Eur J Pediatr. 1992 Apr;151(4):300-3. doi: 10.1007/BF02072234. PMID: 1499584.	Case report/ series	Review search
Meyer CL, Payne NR, Roback SA. Spontaneous, isolated intestinal perforations in neonates with birth weight less than 1,000 g not associated with necrotizing enterocolitis. J Pediatr Surg. 1991 Jun;26(6):714-7. doi: 10.1016/0022-3468(91)90017-n. PMID: 1941464.	Case report/ series	Review search
Borzotta AP, Groff DB. Gastrointestinal perforation in infants. Am J Surg. 1988 Mar;155(3):447-52. doi: 10.1016/s0002-9610(88)80111-9. PMID: 3344910.	Case report/ series	Review search
Aschner JL, Deluga KS, Metlay LA, Emmens RW, Hendricks-Munoz KD. Spontaneous focal gastrointestinal perforation in very low birth weight infants. J Pediatr. 1988 Aug;113(2):364-7. doi: 10.1016/s0022-3476(88)80285-3. PMID: 3397802.	Case report/ series	Review search
Litwin A, Avidor I, Schujman E, Grunebaum M, Wilunsky E, Wolloch Y, Reisner SH. Neonatal intestinal perforation caused by congenital defects of the intestinal musculature. Am J Clin Pathol. 1984 Jan;81(1):77-80. doi: 10.1093/ajcp/81.1.77. PMID: 6691305.	Case report/ series	Review search
Ghanem, S., et al. (2010). "Effect of oral ibuprofen on patent ductus arteriosus in premature newborns." J Saudi Heart Assoc **22**(1): 7-12.	non randomized control trial	Removed during analysis
